# Engineering nanoscale H supply chain to accelerate methanol synthesis on ZnZrO_*x*_

**DOI:** 10.1038/s41467-023-36407-1

**Published:** 2023-02-13

**Authors:** Kyungho Lee, Paulo C. D. Mendes, Hyungmin Jeon, Yizhen Song, Maxim Park Dickieson, Uzma Anjum, Luwei Chen, Tsung-Cheng Yang, Chia-Min Yang, Minkee Choi, Sergey M. Kozlov, Ning Yan

**Affiliations:** 1 Department of Chemical and Biomolecular Engineering, National University of Singapore, 4 Engineering Drive 4, Singapore, 117585 Singapore; 2 Department of Chemical and Biomolecular Engineering, KAIST, Daejeon, 34141 Republic of Korea; 3 Institute of Sustainability for Chemical, Energy and Environment, Agency for Science, Technology and Research (A*STAR), Singapore, 627833 Singapore; 4 Department of Chemistry, National Tsing Hua University, Hsinchu, 300044 Taiwan; 5 Frontier Research Center on Fundamental and Applied Sciences of Matters, National Tsing Hua University, Hsinchu, 300044 Taiwan

**Keywords:** Catalysis, Chemical engineering

## Abstract

Metal promotion is the most widely adopted strategy for enhancing the hydrogenation functionality of an oxide catalyst. Typically, metal nanoparticles or dopants are located directly on the catalyst surface to create interfacial synergy with active sites on the oxide, but the enhancement effect may be compromised by insufficient hydrogen delivery to these sites. Here, we introduce a strategy to promote a ZnZrO_*x*_ methanol synthesis catalyst by incorporating hydrogen activation and delivery functions through optimized integration of ZnZrO_*x*_ and Pd supported on carbon nanotube (Pd/CNT). The CNT in the Pd/CNT + ZnZrO_*x*_ system delivers hydrogen activated on Pd to a broad area on the ZnZrO_*x*_ surface, with an enhancement factor of 10 compared to the conventional Pd-promoted ZnZrO_*x*_ catalyst, which only transfers hydrogen to Pd-adjacent sites. In CO_2_ hydrogenation to methanol, Pd/CNT + ZnZrO_*x*_ exhibits drastically boosted activity—the highest among reported ZnZrO_*x*_-based catalysts—and excellent stability over 600 h on stream test, showing potential for practical implementation.

## Abstract (web summary)

Boosting activity of oxide catalysts is a long-lasting challenge to developing efficient catalysts for industrially important reactions such as CO_2_-to-methanol. Here, the authors report a strategy for enhancing the activity of a ZnZrO_*x*_ methanol synthesis catalyst via engineered nanoscale H supply.

## Introduction

Aiming to mitigate anthropogenic CO_2_ and utilize it as a valuable product, CO_2_ hydrogenation to methanol has received extensive interest from academia and industry^1–5^. Carbon Recycling International established the world’s first large-scale CO_2_-to-methanol plant using Cu/ZnO/Al_2_O_3_, a classical catalyst originally developed and optimized for syngas-to-methanol conversion^6^. Numerous efforts are still being undertaken to elucidate the active site and optimize catalyst activity^7–10^. Nevertheless, one of the critical limitations of Cu/ZnO/Al_2_O_3_ is deactivation due to the unsatisfactory stability of the catalyst under exposure to heat and moisture, hampering long-term process operation^11–14^. Consequently, several Cu-free metal oxides have been reported as new classes of catalysts for CO_2_ hydrogenation to methanol^15–19^, among which, ZnO-ZrO_2_ solid solution catalyst (ZnZrO_*x*_) reported by Li and co-workers^18^ is undergoing pilot-scale testing^20^. One distinctive feature of ZnZrO_*x*_ is its superior long-term stability; however, a downside is its lower activity compared to Cu/ZnO/Al_2_O_3_. Achieving both high activity and long-term stability is a challenging mission for developing next-generation CO_2_ hydrogenation catalysts.

Since H_2_ activation over metal oxide catalysts is often kinetically limited^16,21,22^, the metal promotion has been widely explored for improving methanol formation activity. In particular, for ZnZrO_*x*_, various metals such as Cu^23^, Pt^23^, Ga^24^, and Pd^22,23,25^ have been reported as effective metal promoters. It is a general belief that the metal promoter and oxide support should be placed close to each other to maximize interfacial synergy (including strong metal-support interaction), and thus, various synthetic techniques were developed to anchor metal promotors directly on the oxide catalyst surface^26–38^. Previously, we studied Pd-promoted ZnZrO_*x*_ catalyst (Pd/ZnZrO_*x*_) for methanol formation^22^, revealing that the major role of Pd is to activate and split H_2_ into H atoms that are subsequently transferred to adjacent Zn sites for methanol synthesis. Thus, the rate of H supply to the active sites was identified as a key factor governing the rate of CO_2_ reduction to methanol. However, at present, there are limited ways to enhance the interfacial area between the Pd promoter and ZnZrO_*x*_ catalyst for traditional metal/oxide nanocomposite catalysts.

Herein, we address this challenge by combining ZnZrO_*x*_ with Pd supported on carbon nanotubes (CNT): Pd/CNT + ZnZrO_*x*_. CNT is a well-known material for hydrogen storage^39–41^, and doping CNT with metal- or metal oxide- greatly enhances its H storage capacity, hinting at swift hydrogen transfer between CNT and the dopants^40–44^. Moreover, Crossley and co-workers conducted an elegant study of the nature of active sites on Pd/TiO_2_ and Cu/TiO_2_ catalysts in furfural and anisole hydrogenations by separating metal from the support by a controlled distance using CNT, further substantiating that CNT may act as a bridge between metal and oxide^45^. Torrente-Murciano and co-workers reported the superiority of graphitized CNT as a support for Ru catalysts in NH_3_ cracking, due to the electronic modification of Ru particles by CNT^46^. Based on these previous reports, and the unique property of CNT, we presumed that Pd/CNT can deliver hydrogen to ZnZrO_*x*_ via hydrogen activation on Pd and spillover via CNT. Our experiments showed that Pd/CNT + ZnZrO_*x*_ features drastically improved methanol formation activity from CO_2_ compared to not only ZnZrO_*x*_, but also Pd/ZnZrO_*x*_ with the same Pd content. The Pd/CNT + ZnZrO_*x*_ additionally exhibits excellent long-term stability, surpassing the performance of the state-of-the-art Cu/ZnO/Al_2_O_3_ industrial catalysts during the on-stream test from 200 h onwards.

## Results

### Preparation and characterization of the catalysts

**Fig. 1:**
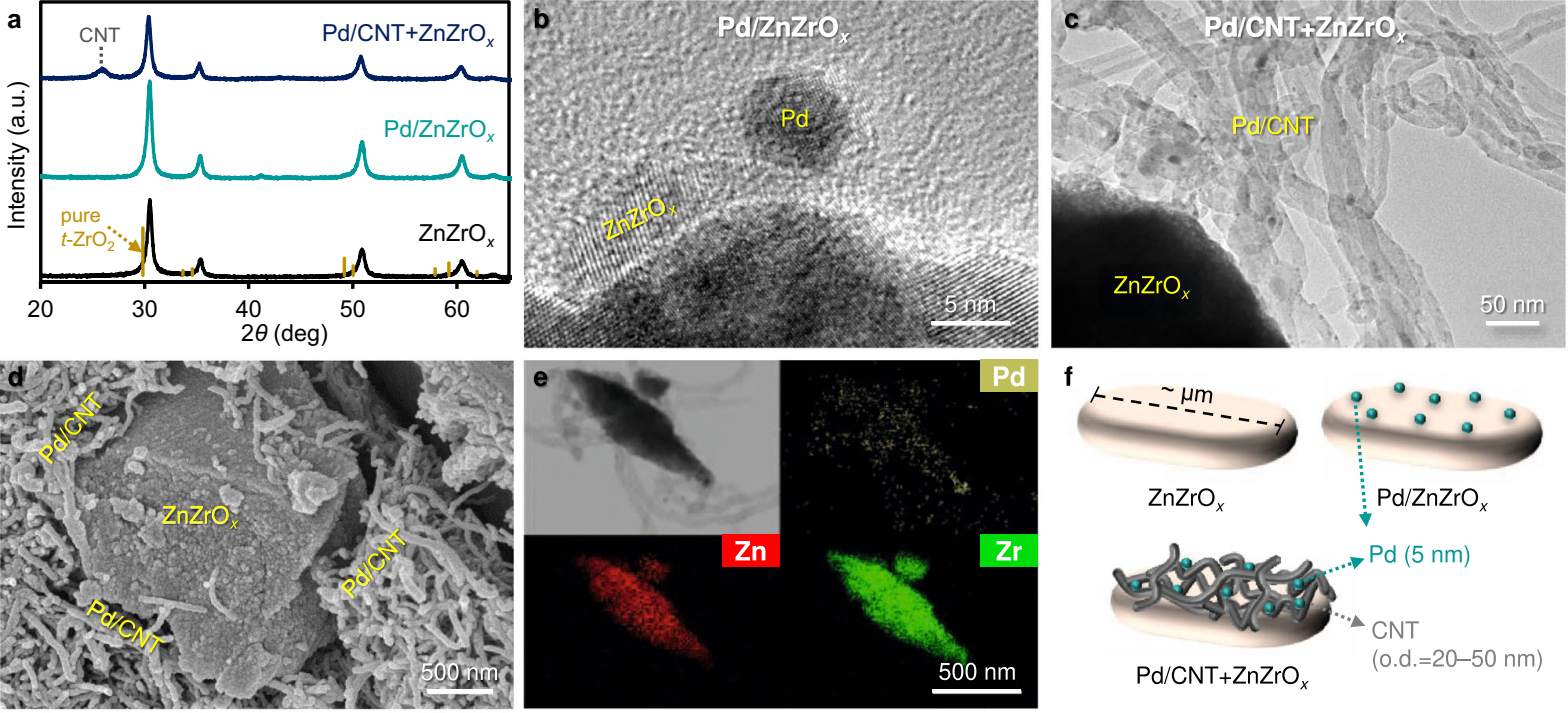
Structural characterization of catalysts. **a** Powder XRD patterns of ZnZrO_*x*_, Pd/ZnZrO_*x*_, and Pd/CNT + ZnZrO_*x*_. The peak shift to higher 2*θ* of ZnZrO_*x*_ compared to pure ZrO_2_ indicates the substitution of Zr atoms to Zn atoms (i.e., solid solution formation). The XRD pattern of pure *t*-ZrO_2_ is obtained from Materials Data on ZrO_2_ by Materials Project, ID: mp-2574. **b** TEM image of Pd/ZnZrO_*x*_. **c** TEM image of Pd/CNT + ZnZrO_*x*_. **d** SEM image of Pd/CNT + ZnZrO_*x*_. **e** EDX elemental maps of Pd/CNT + ZnZrO_*x*_ during TEM analysis. **f** Illustration of the structures for ZnZrO_*x*_, Pd/ZnZrO_*x*_, and Pd/CNT + ZnZrO_*x*_ catalysts (o.d. outer diameter).

The structures of ZnZrO_*x*_, Pd/ZnZrO_*x*_, and Pd/CNT + ZnZrO_*x*_ catalysts were characterized using various techniques. According to TEM and SEM analyses, ZnZrO_*x*_ is composed of *ca*. 10–15 nm crystallites, which are present as agglomerated ~µm-scale particles (Supplementary Fig. 1). Pd/CNT contains uniformly dispersed Pd nanoparticles (*ca*. 5 nm) inside and outside the carbon tubes. (Supplementary Fig. 2). XRD analysis proves that ZnZrO_*x*_ has a *t*-ZrO_2_ phase-like crystalline solid solution structure^18^. The crystallinity of ZnZrO_*x*_ is well-preserved after Pd-impregnation as well as physical mixing with Pd/CNT (Fig. 1a). The TEM image of Pd/ZnZrO_*x*_ shows the presence of *ca*. 5 nm Pd nanoparticles on the surface of ZnZrO_*x*_ (Fig. 1b). In Pd/CNT + ZnZrO_*x*_, the majority of Pd particles are present far away from the ZnZrO_*x*_ surface (Fig. 1c). As Pd/CNT and ZnZrO_*x*_ entangle with each other (Fig. 1c, d), EDX elemental mapping in the 2D-projection shows a uniform distribution of Pd over the catalyst domain (Fig. 1e). H_2_- and CO-chemisorption analysis confirms that Pd/ZnZrO_*x*_ and Pd/CNT have a similar Pd dispersion of 18–20%, which is consistent with TEM analysis. Of note, Pd dispersion is not altered during physical mixing (Supplementary Table 1). Based on these results, we present an illustration of the three catalysts in Fig. 1f. Furthermore, Pd/GNP (GNP: graphene nanoplatelets), Pd/AC (AC: activated carbon), Pd/SiO_2_ and Pd/TiO_2_ were prepared and mixed with ZnZrO_*x*_ as control samples. The characterizations of these catalysts are included in Supplementary Figs. 3, 4 and Supplementary Table 1. The general observations about their structural features are very similar to those of Pd/CNT and Pd/CNT + ZnZrO_*x*_.

### Unique CO_2_ hydrogenation performance of Pd/CNT + ZnZrO_*x*_

The activities of ZnZrO_*x*_ and various Pd-promoted ZnZrO_*x*_ catalysts for CO_2_ hydrogenation to methanol (MeOH) were investigated under the reaction kinetics-controlled region (conditions: 533 K, 5 MPa total pressure with CO_2_/H_2_/Ar = 19/76/5 (molar), conversion: 0.64–1.36%). The exclusion of external and internal mass transfer limitation is described in Section 3 in Supplementary Information.

**Fig. 2:**
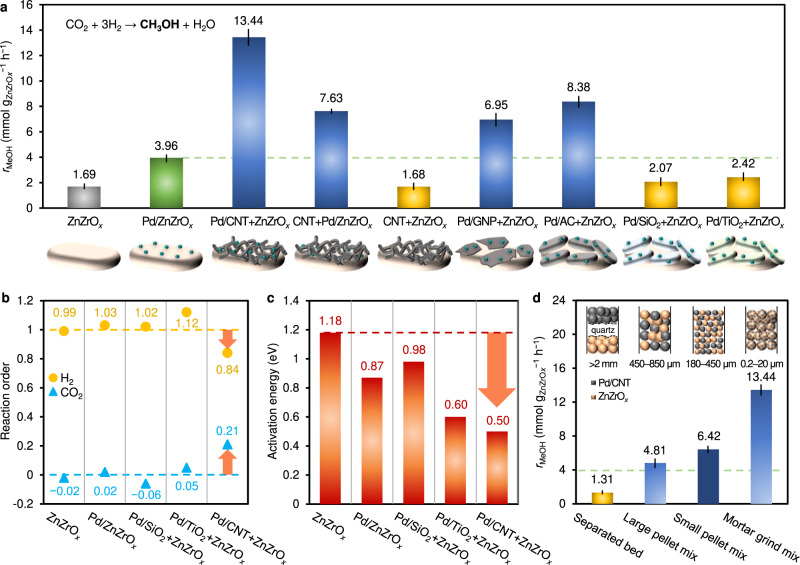
Analysis of reaction kinetics of ZnZrO_*x*_ and Pd-promoted ZnZrO_*x*_ catalysts in the CO_2_ hydrogenation to methanol. **a** Methanol formation rate (*r*_MeOH_) over ZnZrO_*x*_-based catalysts (Condition: 533 K, 5 MPa, CO_2_/H_2_/Ar = 19/76/5 (molar), gas hourly space velocities (GHSV) are shown in Supplementary Table 2). **b** Reaction orders with respect to H_2_ and CO_2_ for methanol formation. **c** Apparent activation energy for methanol formation. The values were determined by the Arrhenius plot shown in Supplementary Fig. 7. **d** Effect of catalyst mixing configurations on methanol formation in the combination of Pd/CNT and ZnZrO_*x*_. Inset shows the illustration of each mixing configuration. The green dashed line in Fig. **a**, **d** indicates the activity of Pd/ZnZrO_*x*_.

CO_2_ conversion and product selectivity of the catalysts are shown in Supplementary Table 2. For all catalysts, the major products are methanol and CO. Only Pd/SiO_2_ + ZnZrO_*x*_ and Pd/TiO_2_ + ZnZrO_*x*_ produced CH_4_ (≤3.40%) and dimethyl ether (DME, ≤0.14%) as side products. Since standalone Pd catalysts have negligible methanol formation activity compared to ZnZrO_*x*_ (Supplementary Fig. 5), the methanol formation rate (*r*_MeOH_) of ZnZrO_*x*_-containing catalysts in Fig. 2a was represented based on the mass of ZnZrO_*x*_. Pd/ZnZrO_*x*_ exhibits higher *r*_MeOH_ (3.96 mmol g_ZnZrO*x*_^−1^ h^−1^) than ZnZrO_*x*_ (1.69 mmol g_ZnZrO*x*_^−1^ h^−1^), illustrating the promotional effect from surface Pd nanoparticles. Unexpectedly, a higher *r*_MeOH_ is observed over Pd/CNT + ZnZrO_*x*_ (13.44 mmol g_ZnZrO*x*_^−1^ h^−1^) compared to Pd/ZnZrO_*x*_, which is an exceptional finding, since the metal promoter (Pd) induces a significantly more pronounced effect when away from the active sites on ZnZrO_*x*_. Considering that Pd/ZnZrO_*x*_ and Pd/CNT + ZnZrO_*x*_ possess similar Pd dispersion and identical Pd loading with respect to ZnZrO_*x*_ (Supplementary Table 1), and that no promotion was observed when ZnZrO_*x*_ is mixed with CNT (CNT + ZnZrO_*x*_), both Pd and CNT are concluded to be essential for the superior catalytic performance of Pd/CNT + ZnZrO_*x*_. A strong promoting effect is also observed when Pd/ZnZrO_x_ is mixed with CNT (i.e., *r*_MeOH_ = 7.63 mmol g_ZnZrO*x*_^−1^ h^−1^ for CNT + Pd/ZnZrO_*x*_) as well as when ZnZrO_*x*_ is mixed with other carbon-supported Pd catalysts (i.e., Pd/GNP + ZnZrO_*x*_, Pd/AC + ZnZrO_*x*_). In contrast, when ZnZrO_*x*_ is mixed with Pd/oxide catalysts such as Pd/SiO_2_ and Pd/TiO_2_ (i.e., Pd/SiO_2_ + ZnZrO_*x*_, Pd/TiO_2_ + ZnZrO_*x*_), there is only slight promotion, demonstrating the unique role of carbon supports. Although TiO_2_ is a well-known reducible oxide for fast hydrogen spillover, Pd/TiO_2_ has an order of magnitude higher CO_2_ hydrogenation activity toward CO compared to other Pd/*support* catalysts (Supplementary Fig. 5). Thus, Pd/TiO_2_ converts CO_2_ and H_2_ by itself rather than acting as a ‘hydrogen dispenser’. These observations indicate promoting performance is related to hydrogen spillover efficiency, which strongly depends on the type of support as hydrogen moves as an H^+^/e^−^ pair^47–49^.

In reaction kinetic analyses (Fig. 2b and Supplementary Fig. 6), the reaction orders with respect to H_2_ (*n*_H2_) and CO_2_ (*n*_CO2_) for methanol synthesis over ZnZrO_*x*_ are *ca*. 1.0 and 0, respectively. The results imply that the surface coverage of H_2_ is much lower than that of CO_2_. The *n*_H2_ = *ca*. 1.0 suggests that H_2_ activation may be a rate-determining step. Pd/ZnZrO_*x*_ catalyst shows similar *n*_H2_ and *n*_CO2_ values to those of pristine ZnZrO_*x*_. In contrast, there is a substantial decrease of *n*_H2_ (0.84) alongside a simultaneous increase of *n*_CO2_ (0.21) for Pd/CNT + ZnZrO_*x*_. The result indicates the coverage of hydrogen on the ZnZrO_*x*_ surface increased considerably through combination with Pd/CNT. As shown in Fig. 2c and Supplementary Fig. 7, Pd/CNT + ZnZrO_*x*_ exhibits drastically decreased apparent activation energy (*E*_a_ = 0.50 eV) compared to ZnZrO_*x*_ (*E*_a_ = 1.18 eV), confirming the immense contribution of physical mixing with Pd/CNT on accelerating reaction kinetics.

For the ZnZrO_*x*_ and Pd/CNT combination, the effect of mixing configuration was studied to gain insight into the origin of promotion (Fig. 2d). Four different mixing configurations were prepared by varying the mixing method, namely separated bed, large pellet mix (size: 450–850 µm), small pellet mix (size: 180–450 µm), and mortar grind mix (size: 0.2–20 µm). The separated bed configuration gives a detrimental effect on methanol formation, implying the promoting effect in Pd/CNT + ZnZrO_*x*_ is not originated from the transport of intermediates in the gas-phase. On the other hand, a higher activity compared to pristine ZnZrO_*x*_ was detected for all other configurations. The promoting effect is strengthened as the contact between the two components (Pd/CNT and ZnZrO_*x*_) increases, reaching the maximum in the mortar grind mix configuration. It is noteworthy that even when the two components are mixed in 180–850 μm-scale, the promoting effect (*r*_MeOH_ = 4.81–6.42 mmol g_ZnZrO*x*_^−1^ h^−1^) is stronger than that of Pd/ZnZrO_*x*_ (Fig. 2a, *r*_MeOH_ = 3.96 mmol g_ZnZrO*x*_^−1^ h^−1^). This result firmly demonstrates the effectiveness of the strategy for hydrogen delivery; hydrogen transfer efficiency is no longer limited by the number of Pd-ZnZrO_*x*_ interface sites, but rather by the closeness of ZnZrO_*x*_ particles and CNT.

We further tested the performance of Pd/CNT + ZnZrO_*x*_ under industry-relevant reaction conditions (5 MPa, GHSV = 24,000 cm^3^_STP_ g_cat._^−1^ h^−1^) to evaluate the practical potential of this catalyst. First, CO_2_ conversion and selectivity of products at different temperatures (513–633 K) are summarized in Supplementary Table 3. The result indicates that methanol and CO are the major products under these reaction conditions. The selectivity of Pd/CNT + ZnZrO_*x*_ is lower than that of ZnZrO_*x*_ and Pd/ZnZrO_*x*_ due to the CO formation activity of Pd/CNT itself (Supplementary Fig. 5). Nevertheless, the MeOH selectivity gap between Pd/CNT + ZnZrO_*x*_ and ZnZrO_*x*_ is less than 10% at the same CO_2_ conversion level (Supplementary Fig. 8).

**Fig. 3:**
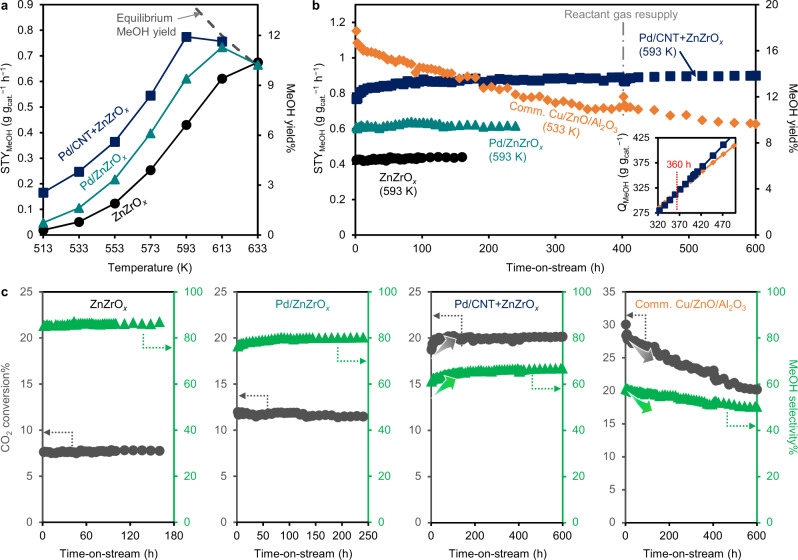
Catalytic performance of Pd/CNT + ZnZrO_*x*_ in industry-relevant conditions. **a** Methanol yield and STY_MeOH_ of the ZnZrO_*x*_-based catalysts as a function of reaction temperature. The dashed line indicates the methanol yield at thermodynamic equilibrium. **b** Long-term catalytic tests of ZnZrO_*x*_*-*based catalysts (593 K), and commercial Cu/ZnO/Al_2_O_3_ (533 K). The inset shows cumulative methanol production (*Q*_MeOH_, g g_cat._^−1^) as a function of time-on-stream (Condition: 5 MPa, CO_2_/H_2_/Ar = 19/76/5, GHSV = 24,000 cm^3^_STP_ g_cat._^−1^ h^−1^). **c** CO_2_ conversion and methanol selectivity changes during long-term reaction test over ZnZrO_*x*_, Pd/ZnZrO_*x*_, Pd/CNT + ZnZrO_*x*_, and commercial Cu/ZnO/Al_2_O_3_ catalysts.

Methanol yield and corresponding space-time yield of methanol (STY_MeOH_) were monitored while varying reaction temperature (Fig. 3a). The STY_MeOH_ for Pd/CNT + ZnZrO_*x*_ is 0.780 g g_cat._^−1^ h^−1^ at 593 K, the highest value among all reported ZnZrO_*x*_-based catalysts (Supplementary Table 4). Moreover, the long-term stability of each catalyst was monitored at 593 K while a commercial Cu/ZnO/Al_2_O_3_ catalyst was simultaneously introduced as a control sample. As shown in Supplementary Fig. 9, the optimal reaction temperature for Cu/ZnO/Al_2_O_3_ catalyst that shows the maximum STY_MeOH_ is 533 K (thermodynamic equilibrium MeOH yield = 24%), thus the long-term test was conducted at this temperature for Cu/ZnO/Al_2_O_3_. Not unexpectedly, Cu/ZnO/Al_2_O_3_ exhibits relatively high activity initially but shows rapid deactivation from the beginning (Fig. 3b); after 600 h of reaction, 46% of initial activity has been lost. In contrast, Pd/CNT + ZnZrO_*x*_ catalyst shows superior long-term performance; the methanol yield over Pd/CNT + ZnZrO_*x*_ reaches 13.5% (STY_MeOH_ = 0.90 g g_cat._^−1^ h^−1^) at 600 h time-on-stream, very close to the value at thermodynamic equilibrium (MeOH yield = 14.0% at 593 K). XANES and EXAFS analyses after the reaction reveal that there are no substantial changes in the chemical state of either Zn or Zr in Pd/CNT + ZnZrO_*x*_, validating the superior stability of Pd/CNT + ZnZrO_*x*_ (Supplementary Figs. 10–11). Both catalysts were additionally tested at a higher GHSV (80,000 cm^3^_STP_ g_cat._^−1^ h^−1^) to shift conversion further away from thermodynamic equilibrium. Again, Cu/ZnO/Al_2_O_3_ exhibited continued deactivation while the Pd/CNT + ZnZrO_*x*_ system kept increasing its methanol productivity during a 104 h test. (Supplementary Fig. 12). Compared to ZnZrO_*x*_ and Pd/ZnZrO_*x*_, distinct increases in conversion and methanol selectivity are observed for Pd/CNT + ZnZrO_*x*_, particularly during the initial stage of reaction (Fig. 3c). Under the steady state (time-on-stream >400 h), Pd/CNT + ZnZrO_*x*_ shows 66% methanol selectivity at 20% CO_2_ conversion, higher than the methanol selectivity of Cu/ZnO/Al_2_O_3_ at comparable CO_2_ conversion (50–52% at 23–20% CO_2_ conversion). As a result of the opposite activity variation trend, cumulative methanol production (*Q*_MeOH_) of Pd/CNT + ZnZrO_*x*_ overwhelms that of commercial Cu/ZnO/Al_2_O_3_ after 360 h reaction (Fig. 3b, inset). At 600 h, Pd/CNT + ZnZrO_*x*_ offered a 1.44-fold higher methanol yield over the commercial Cu/ZnO/Al_2_O_3_ catalyst, and its STY_MeOH_ (g g_cat._^−1^ h^−1^) compare favorably with state-of-the-art catalysts, that were scrutinized in long-term stability tests in the literature (Supplementary Table 5), further validating the superior performance of the Pd/CNT + ZnZrO_*x*_ catalyst. In addition, Pd/CNT + ZnZrO_*x*_ contains 33 wt% CNT, which can act as a catalyst binder/diluent due to its high mechanical strength^50^ and thermal conductivity^51^. Therefore, Pd/CNT + ZnZrO_*x*_ may not require further use of an extra binder/diluent, providing additional benefits for practical implementation.

### Characterization of the promotion effect in Pd/CNT + ZnZrO_*x*_

**Fig. 4:**
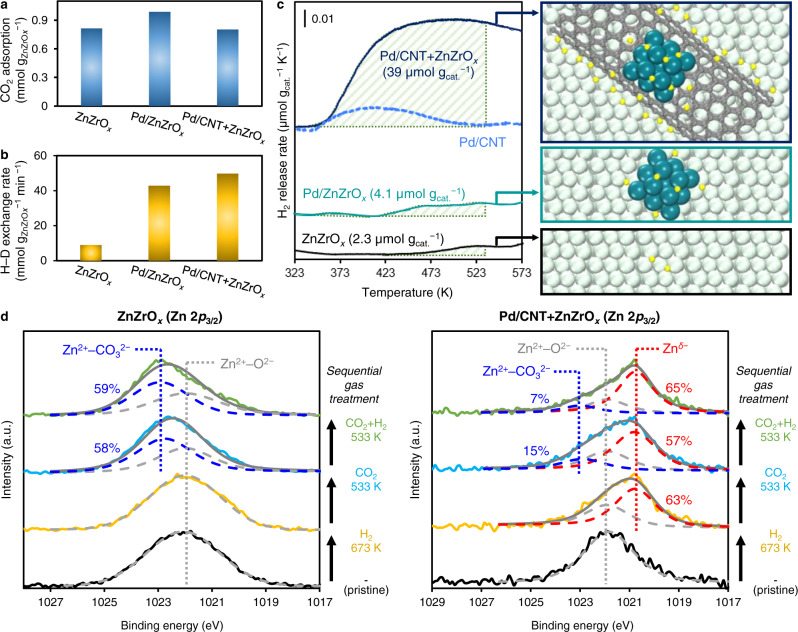
Quantitative gas adsorption-desorption analysis of catalysts. **a** CO_2_ adsorption capacity of catalysts determined by CO_2_-TPD. **b** H–D exchange rate of catalysts at 373 K. **c** H_2_-TPD profile and quantification of reversible H_2_ of catalysts. The quantification of reversible H_2_ was conducted by integrating the peak area in the range of 323–533 K which is below the temperature of our reaction kinetic analysis. **d** Zn 2*p*_3/2_ XPS of ZnZrO_*x*_ and Pd/CNT + ZnZrO_*x*_ catalysts before and after sequential gas treatments (gas treatment condition: H_2_ at 673 K, CO_2_ at 533 K, and CO_2_ + H_2_ (1:4) at 533 K).

The origin of promotion was elucidated by quantitative gas adsorption-desorption analyses. CO_2_-TPD profiles reveal ZnZrO_*x*_, Pd/ZnZrO_*x*_, and Pd/CNT + ZnZrO_*x*_ have comparable adsorption strengths (Supplementary Fig. 13) and capacity of CO_2_ (Fig. 4a), indicating CO_2_ adsorption properties of the catalysts are similar. H_2_–D_2_ isotope exchange experiments were then conducted at 373 K (Fig. 4b) to determine their H_2_ activation function. ZnZrO_*x*_ shows a low H–D exchange rate (8.93 mmol g_ZnZrO*x*_^−1^ min^−1^), while Pd/ZnZrO_*x*_ and Pd/CNT + ZnZrO_*x*_ show accelerated rates at 42.9 and 49.8 mmol g_ZnZrO*x*_^−1^ min^−1^, respectively. We draw two major conclusions from these isotope exchange experiments. First, Pd species easily activate H_2_, regardless of whether Pd nanoparticles are directly supported on ZnZrO_*x*_, or on CNT. Second, the H_2_ activation function alone is not the critical factor in determining methanol formation activity, since the H–D exchange rates on Pd/ZnZrO_*x*_ and Pd/CNT + ZnZrO_*x*_ are comparable despite their pronounced difference in methanol production.

Next, H_2_-TPD profiles of various catalysts were examined to differentiate their ability in delivering activated hydrogen (H*) (Fig. 4c). ZnZrO_*x*_ shows an H_2_ desorption peak above 423 K, while Pd-containing catalysts exhibit a peak starting from 343 K due to more facile desorption of H_2_ from the Pd surface. The amounts of adsorbed H_2_ on ZnZrO_*x*_ and Pd/ZnZrO_*x*_ were 2.3 and 4.1 µmol g_cat._^−1^, respectively. In sharp contrast, Pd/CNT + ZnZrO_*x*_ shows an order of magnitude increase in H_2_ adsorption (39 µmol g_cat._^−1^). Considering Pd/CNT alone only possesses 7.1 µmol H_2_ g_cat._^−1^, and that Pd/CNT accounts for one-third of the mass in the mixture catalyst, over 90% of the adsorbed hydrogen in the Pd/CNT + ZnZrO_*x*_ mixture is stored at the interfaces between ZnZrO_*x*_ and CNT, which undergo reverse spillover during TPD analysis (Fig. 4c, right panels). In the case of Pd/SiO_2_ and Pd/TiO_2_, there are only marginal increments in the H_2_ adsorption capacity after physical mixing with ZnZrO_*x*_ (Supplementary Fig. 14), confirming the unique property of CNT as a hydrogen delivery system. Band gap analysis suggests carbonaceous materials have much smaller band gap values (0.74–0.75 eV) compared to metal oxides (≥3.09 eV) including ZnZrO_*x*_ (Supplementary Fig. 15), suggesting higher electrical conductivity results in faster migration kinetics of hydrogen (H^+^/electron pair) on the carbon surface versus oxides such as ZnZrO_*x*_.

X-ray photoelectron spectroscopy (XPS) after sequential gas treatments (without sample exposure to air) for ZnZrO_*x*_ and Pd/CNT + ZnZrO_*x*_ were carried out to provide more insight into the hydrogen spillover. The spectra of each element are demonstrated in Supplementary Figs. 16–20, while the XPS of Zn 2*p*_3/2_ region is represented in Fig. 4d. Before gas treatment, ZnZrO_*x*_ and Pd/CNT + ZnZrO_*x*_ show identical symmetric Zn 2*p*_3/2_ signals at 1022 eV which represent Zn^2+^ bound to lattice oxygen (Zn^2+^–O^2−^). After H_2_ treatment at 673 K, ZnZrO_*x*_ shows no signal changes, whereas Pd/CNT + ZnZrO_*x*_ exhibits the formation of a shoulder peak at ~1 eV lower binding energy region. The transformation of Zn^2+^–O^2−^ to reduced Zn species (Zn^*δ-*^)^52,53^ is a result of electron transfer from spilt over hydrogen to the Zn site of the ZnZrO_*x*_ surface. Both H_2_-reduced catalysts were further treated with CO_2_ and CO_2_ + H_2_ after which a shoulder peak at ~1 eV higher binding energy region appears, indicative of CO_2_ chemisorption on Zn–O pair (Zn^2+^–CO_3_^2−^)^53^. Notably, the existence of Zn^*δ-*^ species is still pronounced in Pd/CNT + ZnZrO_*x*_ after consecutive treatments with CO_2_ and CO_2_ + H_2_. The portion of Zn^*δ-*^ is slightly decreased after CO_2_ treatment (from 63 to 57%), but it recovers the original fraction when treated with CO_2_ + H_2_ (65%), providing evidence of effective hydrogen spillover in the Pd/CNT + ZnZrO_*x*_ system under CO_2_ hydrogenation condition.

### Computational studies on hydrogen spillover in Pd/CNT + ZnZrO_*x*_

**Fig. 5:**
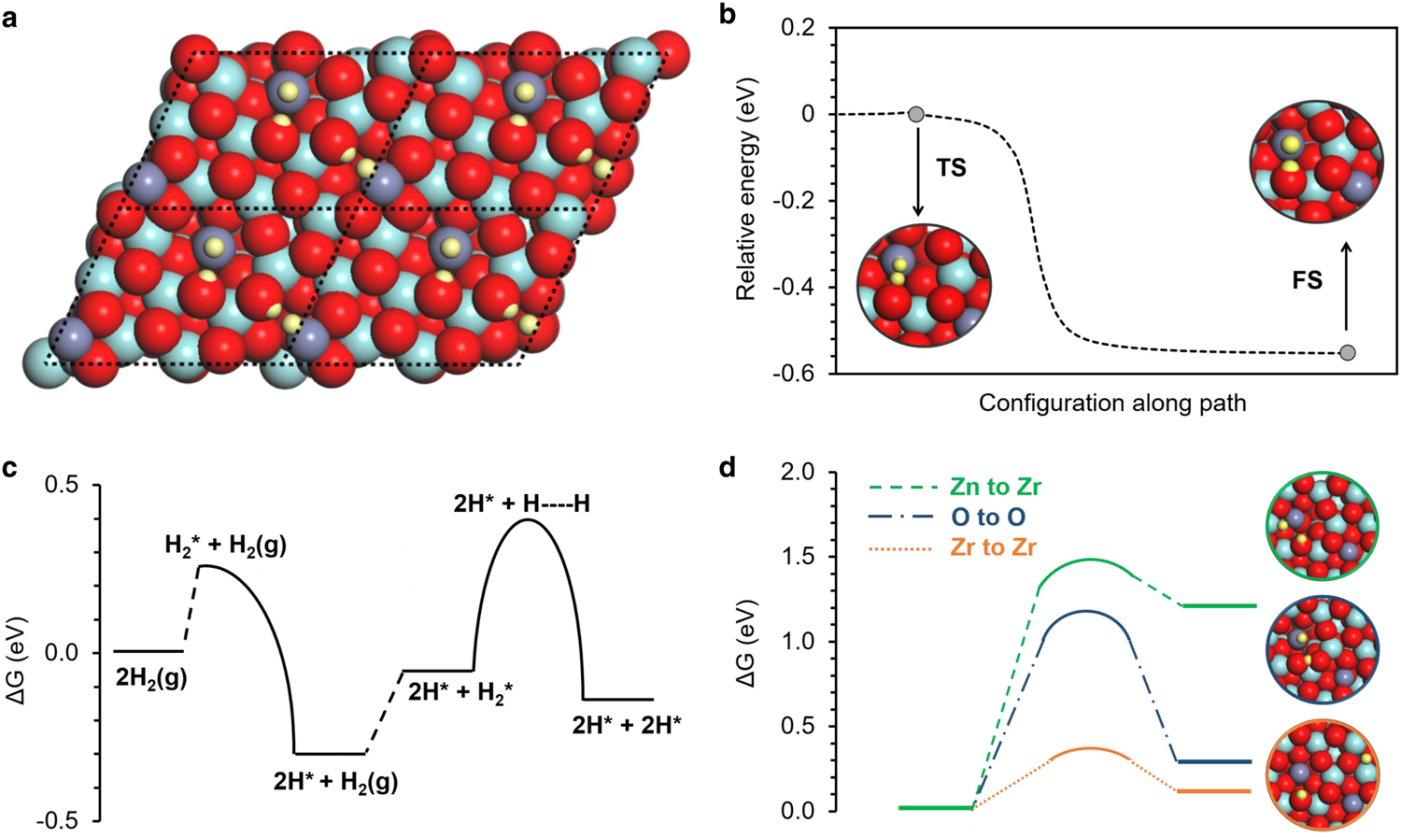
Simulations of H atoms on the bare ZnZrO_*x*_ surface. **a** Top view of four employed ZnZrO_*x*_ supercells illustrating 4 H atoms adsorbed per unit cell on Zn–O pairs, formed after successive dissociation of two H_2_ molecules. **b** Potential energy profile along the reaction coordinate for barrierless dissociation of the first H_2_ molecule (TS transition state, FS final state). **c** Potential energy profile of successive H_2_ dissociation at ZnZrO_*x*_ for 2 H_2_ molecules. **d** Energy profile for H movement from Zn to Zr, O to O, and Zr to Zr on the ZnZrO_*x*_ surface. The inset images show the transition state configurations. Turquoise, purple, red, and yellow colors represent Zr, Zn, O, and H, respectively.

Density functional theory calculations were applied to provide an atomistic description of hydrogen spillover in the Pd/CNT + ZnZrO_*x*_ nanocomposite. After identifying the most stable model for ZnZrO_*x*_ (Supplementary Figs. 21–24), we studied the adsorption and movement of H on the bare ZnZrO_*x*_ surface (Fig. 5 and Supplementary Fig. 25). H_2_ dissociation on pure ZrO_**2**_ is kinetically limited^54^, which can be improved by doping the oxide with Zn atoms^18,55^. We calculated Zn–O pairs in the ZnZrO_*x*_ surface to have the lowest dissociative adsorption energies of H_2_ among all surface sites (Supplementary Fig. 25b). Since our ZnZrO_*x*_ supercell contains two Zn sites (Fig. 5a), we are able to perform simulations for the sequential dissociation of two H_2_ molecules. The dissociation of the first H_2_ molecule is a barrierless step (Fig. 5b) with reaction energy of ΔG = − 0.31 eV (Fig. 5c). Yet, the dissociation of the second H_2_ molecule is impaired by a higher activation barrier (ΔG^a^ = 0.71 eV) and reaction energy of ΔG = 0.17 eV (Fig. 5c), indicating limited H coverage on unmodified ZnZrO_*x*_ under reaction conditions.

Moreover, H atoms are calculated to have negligible mobility on the ZnZrO_*x*_ surface with barriers up to ΔG^a^ = 1.18 eV for diffusion between O sites and ΔG^a^ = 1.51 eV for migration of H atoms from Zn to Zr atoms (Fig. 5d). Although a much lower barrier of ΔG^a^ = 0.34 eV was calculated for H diffusion between Zr sites, these sites were calculated to be up to 2.22 eV less stable for H than O sites on ZnZrO_*x*_ surface based on the adsorption energy of one H atom. These suggest that directly supported Pd nanoparticles cannot deliver reactive H atoms to the active sites on ZnZrO_*x*_ since long-distance H spillover is hindered by high diffusion barriers of H atoms on the oxide surface.

**Fig. 6:**
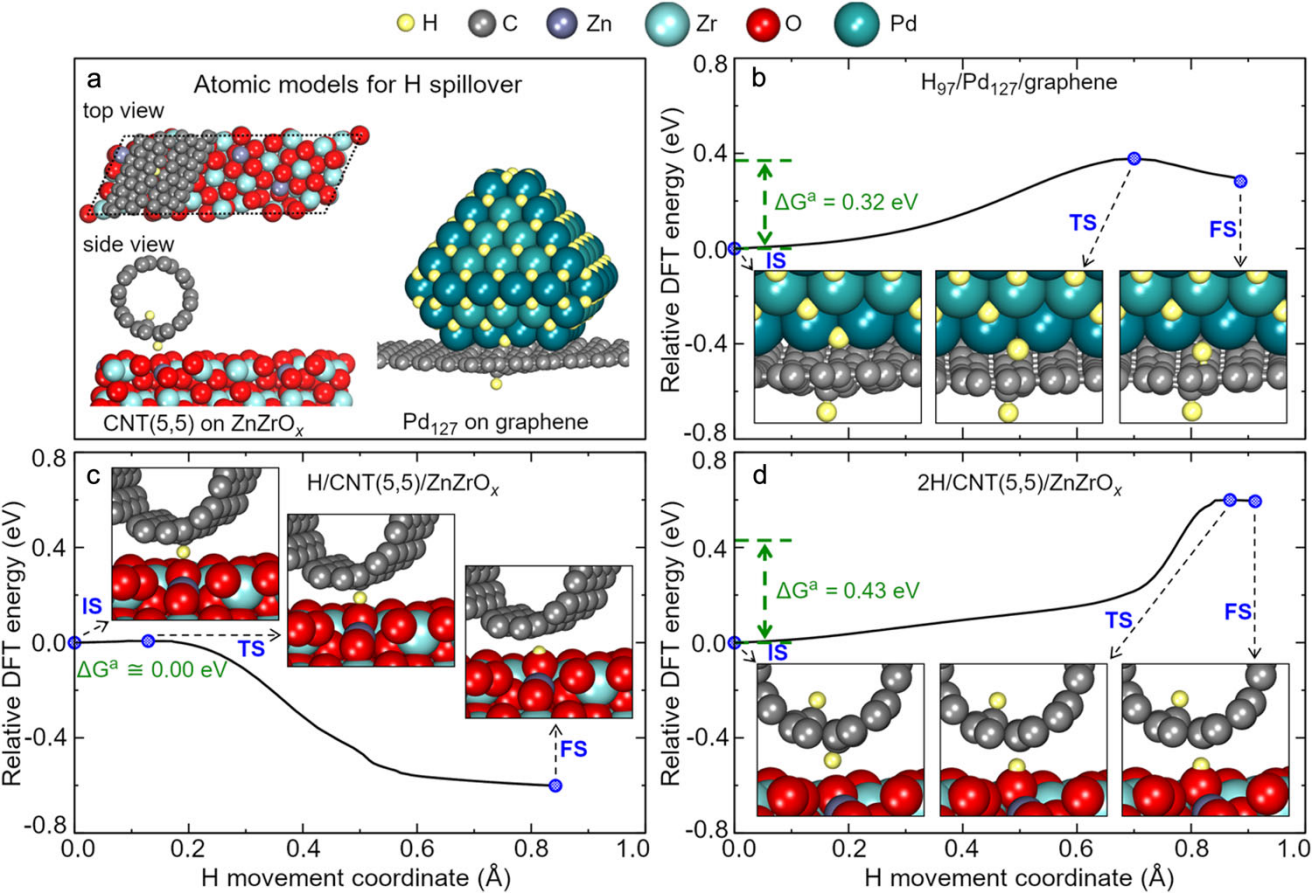
Simulations of hydrogen spillover in Pd/CNT + ZnZrO_*x*_. **a** Atomic models of CNT(5,5) on ZnZrO_*x*_ and Pd on carbon (a graphene sheet) for H spillover. **b** Energy profile for movement of one H from hydrogen-saturated Pd to the graphene sheet with a pre-adsorbed H atom (IS initial state, TS transition state, and FS final state). **c** Energy profile for spillover of the single H atom from CNT(5,5) to ZnZrO_*x*_. **d** Energy profile for spillover of one H atom from CNT(5,5) with another H atom adsorbed on it to ZnZrO_*x*_.

In contrast to the bare ZnZrO_*x*_, our calculations show that H can be fluently supplied to the active sites on the ZnZrO_*x*_ surface via spillover from Pd/CNT (Fig. 6). We created models of physisorbed CNT on ZnZrO_*x*_ using an adapted lattice matching algorithm, which ensured the minimal strain of −2.33% along the CNT length (Fig. 6a). According to our simulations, a single H atom bind stronger to ZnZrO_*x*_ than to CNT, which creates a significant thermodynamic driving force for the spillover and makes this process essentially barrierless (Fig. 6c). However, the spillover barrier increases when more H atoms are adsorbed on CNT. For example, the Gibbs barrier for the spillover of an H atom from CNT to ZnZrO_*x*_ increases to 0.43 eV when another H atom is adsorbed in the most stable position on the vicinal site on the inner side of the nanotube (Fig. 6d). In turn, H spillover from CNT to ZnZrO_*x*_ becomes endothermic by about ΔG ~ 2.5 eV when every C atom of CNT is hydrogenated. In conclusion, low H coverage on CNT can be regarded as the critical structural factor enabling hydrogen spillover from C to the oxide. Low H coverage on CNTs is also suggested by our XPS studies showing mostly *sp*^2^ hybridization of C atoms in the CNT under reaction conditions (Supplementary Fig. 19).

To evaluate the rate of H spillover from Pd to *sp*^2^ carbon, we simulated the movement of H from a 1.56 nm large Pd_127_ fcc nanocrystallite to a graphene sheet representing a broad nanotube. We chose the shape of the Pd_127_ particle in line with TEM data and identified the most stable structure of the Pd_127_/graphene interface by screening all high-symmetry interfaces (Supplementary Fig. 26). Our calculations show that the barriers for H spillover from Pd to graphene are essentially governed by the endothermicity of this process (Fig. 6b), which depends on the H coverage. Namely, the spillover of the first H atom from Pd_127_ to graphene is endothermic by ΔG = 1.57 eV, which decreases to ΔG = 0.37 eV when another H atom is present on the graphene sheet. Since the Gibbs adsorption energy per H atom of one (two) H on the freestanding CNT(5,5) is 1.04 eV (0.70 eV/H atom), which is 0.84 eV (0.37 eV/H atom) stronger than on graphene, the curvature of the CNT should further favor the transfer of H from Pd to the carbon domain. Thus, the barriers for H spillover from Pd to CNT are estimated to be much lower than the activation energy of H_2_ dissociation on ZnZrO_*x*_ (ΔG^a^ = 0.71 eV). In turn, CNTs are well known to be excellent transport channels for H atoms^45^, whose movement over graphene was shown to be enabled by quantum tunneling^56^. Thus, our simulations suggest a much faster H supply to the active sites on Pd/CNT + ZnZrO_*x*_ nanocomposite compared to the pristine ZnZrO_*x*_.

### Effect of catalyst structure on catalytic results in CO_2_-to-methanol

**Fig. 7:**
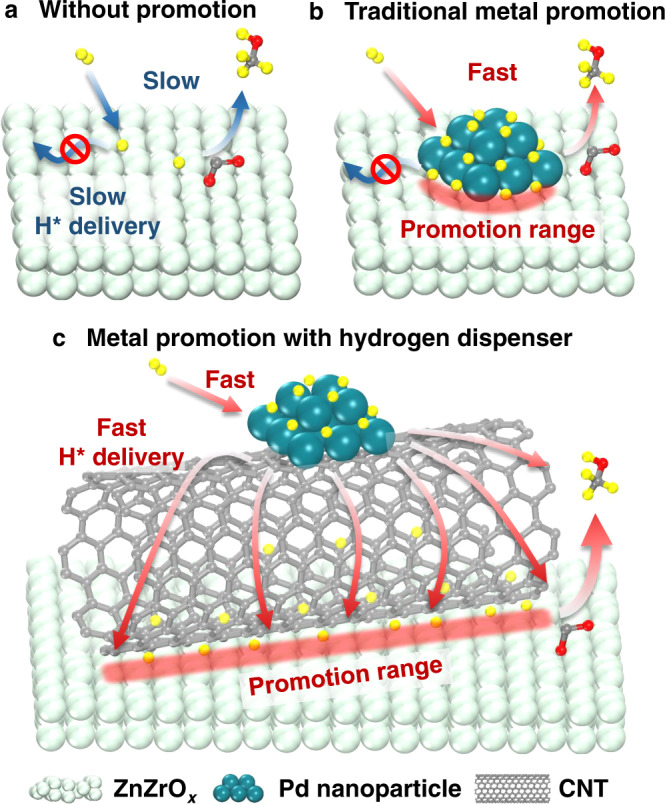
Schematic illustration of the effect of catalyst structure on CO_2_ hydrogenation to methanol. **a** ZnZrO_*x*_ catalyst without any metal promotion. **b** Pd-supported ZnZrO_*x*_ catalyst which represents the traditional metal/oxide catalyst harnessing metal promotion. **c** Pd/CNT + ZnZrO_*x*_ catalyst which represents a conceptually new catalyst system harnessing metal promotion with a hydrogen dispenser.

A number of studies using metal/support catalysts (e.g., Cu or Pd on ZnO, ZrO_2_, or ZnZrO_*x*_) refer to hydrogen spillover as an important process for methanol synthesis from CO/CO_2_^23,57–63^_,_ but it is difficult to disentangle catalytic activity generated by sites adjacent to metals from that generated by sites farther away^64^. In Fig. 7, we present the effect of the catalyst structure on CO_2_ hydrogenation to methanol over Pd-promoted ZnZrO_*x*_ catalysts. ZnZrO_*x*_ is an excellent material to chemisorb CO_2_, but methanol formation is limited due to low H_2_ activation and H* delivery capability (Fig. 7a). In turn, Pd particles supported on ZnZrO_*x*_ significantly enhance H_2_ activation, but the delivery of H* toward the active sites is hampered due to slow H-migration kinetics within ZnZrO_*x*_ surface (Fig. 7b). It is estimated the contact area between Pd particles and ZnZrO_*x*_ is about 0.5 m^2^ g_cat._^−1^, only 1% of the entire catalyst surface area (estimation procedure available in Section 4 in Supplementary Information). Our experimental and computational studies support Prins’s rigorous critique that hydrogen may not travel farther than the immediate interface between metal and basic oxide support^47^, and Li et al.’s hypothesis that the rate-determining step of CO_2_ hydrogenation to methanol over various catalysts (i.e., ZnZrO_*x*_, Cu/ZnO/Al_2_O_3_, and Pd/ZnO) is the migration of H* on the catalyst surface^65^. In the case of Pd/CNT + ZnZrO_*x*_, CNT acts as a hydrogen dispenser, effectively delivering activated H* from Pd particles to the ZnZrO_*x*_ surface (Fig. 7c). Since the promotion range is determined by the contact area between CNT and ZnZrO_*x*_, a ten-times larger promotion area is generated (as determined by H_2_-TPD result) under mortar grind mode compared to traditional metal doping on the catalyst surface, leading to a drastically enhanced methanol formation rate. In the literature, non-contact integration of multiple catalytically active components, especially physical mixing, has been adopted in catalyst development for bifunctional- or cascade catalytic reactions for which each catalyst is responsible for one step of the entire transformation^66–74^. Our work, in contrast, reports a bicomponent catalyst in which one component provides active sites for the formation of a target product while the other serves as an activation and delivery system for a kinetically hindered intermediate, which to our knowledge, has not been demonstrated for CO_2_ hydrogenation.

Furthermore, our preliminary tests of physical mixing of commercial Cu/ZnO/Al_2_O_3_ with CNT results in 1.4 to 2.0-times higher methanol formation activity versus pristine Cu/ZnO/Al_2_O_3_ in the pressure range of 0.1–1.0 MPa (Supplementary Fig. 27), suggesting this promotion strategy is not limited to ZnZrO_*x*_. For the successful implementation of the CO_2_-to-MeOH process on large scales, recycling of the product stream (including unconverted CO_2_, H_2_, and produced CO) is necessary to intensify process economics^75,76^. Future research should be directed to studying the effect of CO on nanoscale H supply in newly developed systems.

## Discussion

Insufficient H_2_ activation has long been known to limit the activity of oxide catalysts, such as ZnZrO_*x*_, for CO_2_ reduction to methanol. In this work, we report CNT as a hydrogen dispenser for enhancing hydrogen delivery to active sites on ZnZrO_*x*_. The combination of Pd/CNT and ZnZrO_*x*_ leads to an exceptional catalytic system for methanol formation, far better than Pd/ZnZrO_*x*_ catalyst, as CNT effectively delivers Pd-activated hydrogen to a broad range of ZnZrO_*x*_ surface sites. The Pd/CNT + ZnZrO_*x*_ shows the highest STY_MeOH_ among reported non-Cu/ZnO-based catalysts to date and is durable over 600 h continuous operation, demonstrating its potential for practical implementation; concurrently, the physical mixing strategy offers a robust and transferable method for material integration to design better catalytic systems. This study provides an example in oxide catalyst design for which the transition metal promoter in non-contact mode induces a greater effect than the transition metal promoter directly attached to the surface of the catalytically active phase, expanding the concept of metal promotion beyond the traditional focus of metal-support interfaces.

## Methods

### Catalysts preparation

ZnO-ZrO_2_ (Zn/Zr = 1/5) solid solution catalyst (ZnZrO_*x*_) was prepared via a coprecipitation method with reference to the earlier literature^22^. An 1 wt% Pd/ZnZrO_*x*_ catalyst was prepared by wet impregnation using Pd(NO_3_)_2_·*x*H_2_O as Pd precursor. To make the Pd/CNT + ZnZrO_*x*_ catalyst, 2 wt% Pd was first supported on CNT by wet impregnation followed by reduction at 673 K, and then Pd/CNT was physically mixed with ZnZrO_*x*_ with Pd:ZnZrO_*x*_ = 1:100 mass ratio. Physical mixing was carried out by mortar grinding, unless otherwise stated. For comparison, various other physically mixed catalysts (Pd/*support* + ZnZrO_*x*_) were also prepared in a similar manner while varying the support material (*support* = type of support used; AC: activated carbon, GNP: graphene nanoplatelet, SiO_2_: silica, TiO_2_: titania P25). CNT + ZnZrO_*x*_ and CNT + Pd/ZnZrO_*x*_ were also prepared by physical mixing of CNT and ZnZrO_*x*_ or Pd/ZnZrO_*x*_. Prior to the characterizations and catalytic reactions, the catalysts were mortar ground. As shown in Section 5 in Supplementary Information, the mortar grinding itself does not significantly affect the surface area and catalytic activity of ZnZrO_*x*_.

### Catalyst characterization

The structures of catalysts were characterized using transmission electron microscopy (TEM) coupled with energy dispersive X-ray (EDX) elemental analysis, scanning electron microscopy (SEM), powder X-ray diffraction (XRD), N_2_ physisorption, H_2_ and CO chemisorption, CO_2_- and H_2_-temperature-programmed desorption (TPD), H_2_–D_2_ isotope exchange, UV-Vis-NIR spectroscopy, and X-ray photoelectron spectroscopy (XPS).

### Catalytic test

CO_2_ hydrogenation was carried out using an automatic multi-channel high-pressure flow reactor designed by PID Eng & Tech. Prior to the reaction, the catalysts were pressed, cracked, and sieved to make pellets. The sieved catalyst pellets (450–850 µm) were loaded into the reactor, and pretreated with H_2_ (40 cm^3^_STP_ min^−1^) at 673 K for 2 h (For Cu/ZnO/Al_2_O_3_ catalysts, pretreatments were conducted at 573 K). After the pretreatment, the reactor was cooled down to a target temperature (493–633 K), and was pressurized with a reaction gas mixture (CO_2_/H_2_/Ar, typically 19/76/5). The outlet gas line was kept at 453 K to prevent condensation of products, and products were analyzed by online gas chromatography (Agilent 8890) equipped with a flame ionization detector (FID) and a thermal conductivity detector (TCD). Agilent HP-PoraplotQ and Restek ShinCarbon were used as columns. Reported data are given as values of time-on-stream at 3 h unless otherwise stated.

### Computational studies

The computational approach was based on spin-unpolarized density functional theory within the Perdew-Burke-Ernzerhof^77^ exchange-correlation approximation as implemented in the Vienna Ab initio simulation package (VASP)^78,79^. The zero-damping D3 correction was added to improve the description of dispersive interactions^80^. To treat strong correlation effects for *d* electrons, the simplified implementation of Hubbard corrections^81^ was adopted with U − J = 4 eV on Zr atoms. Atomic charges were assigned using Bader charge analysis^82,83^. The Gibbs adsorption energies of H were calculated as ${G}_{{ad}}=G[{{{{{\rm{H}}}}}}/{{{{{\rm{cat}}}}}}]-E[{{{{{\rm{cat}}}}}}]-0.5\,G[{{{\mbox{H}}}}_{2}]$, where, $G[{{{{{\rm{H}}}}}}/{{{{{\rm{cat}}}}}}]$ is the Gibbs total energy of the system with the adsorbate, while $E[{{{{{\rm{cat}}}}}}]$ and $G[{{{{{{\rm{H}}}}}}}_{2}]$ are the total and Gibbs total energy of the isolated catalyst model and H_2_ molecule in the gas phase. All energetic properties except for the relative energies in Figs. 5b and 6b–d, include Gibbs corrections calculated at 533 K and 4 MPa of H_2_ pressure under either the ideal gas or harmonic approximations^84,85^. In Fig. 6, the H movement coordinate is the quadratic distance of each structure with respect to the corresponding initial state. The **k**-point sampling was performed using Monkhorst-Pack meshes yielding converged adsorption energies of H, namely, the **k**-mesh parameters were 8 × 1 × 1 for all systems containing CNT(5,5), 2 × 2 × 1 for the 10×10 supercell of graphene, while the gamma point was sufficient for calculations involving only the ZnZrO_*x*_ supercell. All models had a vacuum region of about 11 Å between adjacent slabs and all lateral distances between periodic images were at least 6 Å to keep interactions between periodic images minimal. All systems were optimized with the convergence criteria of 0.03 eV/Å and 10^−5^ eV for the force on each atom and total electronic energy. To optimize transition states, the climbing image nudged elastic band^86,87^ and dimer^88^ methods were employed and the optimized first-order saddle points were confirmed to have one imaginary vibrational frequency leading to the reported local minima.

Additional details of the methods for catalyst preparation, catalyst characterization, catalytic test, and computational studies are provided in the Supplementary Information.

## Supplementary information

Supplementary Information

## Data Availability

Relevant data supporting the key findings of this study are available within the article and the Supplementary Information file. All raw data generated during the current study are available from the corresponding authors upon request.
