# Supplementary material for: Engineering nanoscale H supply chain to accelerate methanol synthesis on ZnZrOx
Source: Nat Commun. 2023 Feb 13;14:819. doi: 10.1038/s41467-023-36407-1 (PMC9925737; doi:10.1038/s41467-023-36407-1)
Supplement: Supplementary file 1 — Supplementary Information [file 41467_2023_36407_MOESM1_ESM.pdf]

Supplementary Information

Engineering nanoscale H supply chain to accelerate methanol synthesis on $ZnZrO_x$

Kyungho Lee,[a] Paulo C. D. Mendes,[a] Hyungmin Jeon,[b] Yizhen Song,[a] Maxim Park Dickieson,[a] Uzma Anjum,[a] Luwei Chen,[c] Tsung-Cheng Yang,[d] Chia-Min Yang,[d,e] Minkee Choi,[b] Sergey M. Kozlov,[a,*] Ning Yan[a,*]

[a] Department of Chemical and Biomolecular Engineering, National University of Singapore, 4 Engineering Drive 4, Singapore, 117585, Singapore

[b] Department of Chemical and Biomolecular Engineering, KAIST, Daejeon, 34141, Republic of Korea

[c] Institute of Sustainability for Chemical, Energy and Environment, Agency for Science, Technology and Research (A*STAR), Singapore, 627833, Singapore

[d] Department of Chemistry, National Tsing Hua University, Hsinchu, 300044, Taiwan

[e] Frontier Research Center on Fundamental and Applied Sciences of Matters, National Tsing Hua University, Hsinchu, 300044, Taiwan

[*] Corresponding authors (cheserg@nus.edu.sg, ning.yan@nus.edu.sg)

Table of Contents

1. **Methods ……………………………………………………………….p. 2**
2. **Supplementary tables and figures ……………………………………….p. 9**
3. **Identify kinetics-controlled region ……………………………………….p. 31**
4. **Estimation of interfacial area of Pd and $ZnZrO_x$ in Pd/$ZnZrO_x$ …… p. 33**
5. **Effects of mortar grinding on $ZnZrO_x$ ………………………………...p. 34**
6. **References………………………………………………………......p. 35**

1. Methods

Materials

$Zn(NO_3)_2{\cdot}6H_2O$ (98%), $ZrO(NO_3)_2{\cdot}xH_2O$ (99%), $(NH_4)_2CO_3$ (≥30% NH_3 basis) and $Pd(NO_3)_2{\cdot}xH_2O$ (40% Pd basis) were purchased from Sigma Aldrich. CNT (multiwalled carbon nanotube, ANR technologies), AC (activated carbon, Sigma Aldrich), and GNP (graphene nanoplatelets, Sigma Aldrich) were used after washing with a 6 M HCl aqueous solution (30 cm^3 g^{-1}) to remove metal impurities, whereas SiO_2 (Davisil grade 633) and TiO_2 (Degussa P25) were used without any treatment.

Catalysts preparation

Synthesis of ZnZrO$_x$. ZnZrO$_x$ solid solution catalyst was prepared by a coprecipitation method. Typically, 10.6 g of $Zn(NO_3)_2{\cdot}6H_2O$ and 11.3 g of $ZrO(NO_3)_2{\cdot}xH_2O$ were dissolved in 140 cm^3 of deionized (DI) water at 343 K. The precipitant, 0.625 M $(NH_4)_2CO_3$ aqueous solution 100 cm^3, was added dropwise to the aforementioned solution under vigorous stirring. The suspension was aged for 2 h at 343 K under vigorous stirring (800 rpm). After cooling down to room temperature, the precipitate was recovered by filtration and washed thoroughly with DI water. The resulting gel was dried at 373 K for 12 h and calcined at 773 K under air flow for 3 h.

Synthesis of Pd/ZnZrO$_x$. 1 wt% Pd was supported on as-prepared ZnZrO$_x$ by incipient wetness impregnation using aqueous $Pd(NO_3)_2{\cdot}xH_2O$ solution. After the evaporation of the solvent at 343 K, the sample was dried at 373 K for 12 h. The dried sample was calcined and reduced at 673 K under dry air and 5%H_2/N_2 flow for 2 h, respectively.

Synthesis of Pd/CNT+ZnZrO$_x$. 2 wt% Pd supported on CNT (Pd/CNT) was prepared by incipient wetness impregnation using aqueous $Pd(NO_3)_2{\cdot}xH_2O$ solution. After the evaporation of the solvent at 343 K, the sample was dried at 373 K for 8 h, followed by reducing at 673 K for 2 h under 5%H_2/N_2 flow. The reduced Pd/CNT was physically mixed with as-prepared ZnZrO$_x$ by mortar grinding with Pd:ZnZrO$_x$ = 1:100 mass ratio (i.e. Pd/CNT:ZnZrO$_x$ = 1:2 mass ratio).

Synthesis of other physically mixed catalysts. Various 2 wt% Pd supported catalysts, Pd/AC, Pd/GNP, Pd/SiO_2, Pd/TiO_2, were prepared by incipient wetness impregnation using aqueous $Pd(NO_3)_2{\cdot}xH_2O$ solution. The evaporation and drying methods are similar to those employed in the synthesis of Pd/CNT. For Pd/AC and Pd/GNP, the dried sample was reduced at 673 K for 2 h under 5%H_2/N_2 flow. For Pd/SiO_2 and Pd/TiO_2, the dried sample was calcined and reduced at 673 K for 2 h under dry air and 5%H_2/N_2 flow, respectively. The reduced Pd/*support* catalysts were physically mixed with as-prepared ZnZrO$_x$ at Pd:ZnZrO$_x$ = 1:100 mass ratio by mortar grinding. CNT+Pd/ZnZrO$_x$ was prepared by mortar grinding of CNT and as-prepared Pd/ZnZrO$_x$ (CNT:Pd/ZnZrO$_x$ = 1:2 mass ratio). CNT+ZnZrO$_x$ was prepared by mortar grinding of CNT and as-prepared ZnZrO$_x$ (CNT:ZnZrO$_x$ = 1:2 mass ratio).

Preparation of Cu/ZnO/Al_2O_3 catalysts. A commercial Cu/ZnO/Al_2O_3 methanol synthesis catalyst was purchased from Alfa Aesar (Product no. 45776). Lab-made Cu/ZnO/Al_2O_3 catalyst was prepared by

coprecipitation of $Cu(NO_3)_2·3H_2O$ (>98%, Sigma Aldrich), $Zn(NO_3)_2·6H_2O$, and $Al(NO_3)_3·9H_2O$ (>98%, Sigma Aldrich) with Na_2CO_3 (anhydrous, Sigma Aldrich) as precipitant. 4.35 g of $Cu(NO_3)_2·3H_2O$, 2.68 g of $Zn(NO_3)_2·6H_2O$, and 1.12 g of $Al(NO_3)_3·9H_2O$ (Cu/Zn/Al = 6/3/1 molar) were dissolved in 100 cm^3 of DI water at 343 K. The precipitant, 0.3 M Na_2CO_3 aqueous solution 120 cm^3, was added dropwise to the aforementioned solution under vigorous stirring. The suspension was aged for 2 h at 343 K under vigorous stirring (800 rpm). After cooling to room temperature, the precipitate was recovered by filtration and washed thoroughly with DI water. The resulting gel was dried at 353 K for 12 h and calcined at 623 K under air flow for 3 h.

Catalyst characterization

Transmission electron microscopy (**TEM**) was performed using a JEM 2100F (JEOL) operating at 200 kV. Prior to the analysis, the powdery samples were dispersed in ethanol (HPLC grade) and placed on a Formvar-coated copper grid. The number-weighted average particle size of Pd was determined by the equation

$d_{\mathrm{Pd}} = \frac{\sum d_i n_i}{\sum n_i}$, where d_i is the particle diameter, and n_i is the number of particles with this diameter. (1)

Energy dispersive X-ray (**EDX)** elemental mapping was conducted in an Oxford instrument during TEM analysis.

Scanning electron microscopy (**SEM**) was performed using a JSM-7610Plus (JEOL) microscope operating at 5 kV and 32.8 μA.

Powder X-ray diffraction (**XRD**) patterns were collected using a D2-Phaser diffractometer equipped with a Cu Kα radiation source (40 kV, 30 mA) and a LYNXEYE detector (Bruker). The data were monitored at a resolution of 0.02 degree and count time of 0.2 s for each point.

N_2 physisorption at 77 K was performed using a NOVAtouch NT 4LX-1 volumetric analyzer (Quantachrome). Prior to the measurement, samples were degassed at 623 K for 3 h under high vacuum. Brunauer–Emmett–Teller surface area (S_{BET}) were determined in the P/P_0 range of 0.10–0.30 of the N_2 adsorption branch.

H_2 and CO chemisorption were performed using a Micromeritics ASAP2020 volumetric analyzer. Prior to the measurement, samples were re-reduced at 673 K for 2 h under H_2 flow, and evacuated under high-vacuum at the same temperature for 2 h. To avoid formation of Pd hydrides, the measurements were performed at 343 K. The amount of chemisorption was estimated by extrapolation of the linear portion of the isotherm (5–30 kPa) and the dispersion of Pd particles was determined by using the stoichiometry factors: Pd/H = 1,[1] and Pd/CO = 0.5.[2]

CO_2 temperature-programmed desorption (**CO_2-TPD**) profiles were recorded using a BELCAT II catalyst analyser equipped with a thermal conductivity detector. Typically, 0.1 g of sample was pretreated under H_2 flow (30 cm^3 min^{-1}) at 673 K. Then, CO_2 (30 cm^3 min^{-1}) was dosed to sample at 323 K for 1 h and the weakly adsorbed CO_2 was removed by He flow (30 cm^3 min^{-1}) for 1 h at the same temperature. For the measurement of the TPD profiles, the temperature was increased up to 673 K (ramp: 10 K min^{-1}) under He flow (30 cm^3 min^{-1}). The adsorbed amount of CO_2 was determined in the temperature range of 323–673 K of the profile.

H_2 temperature-programmed desorption (**H_2-TPD**) profiles were measured similarly with CO_2-TPD changing the adsorbate to H_2. The adsorbed amount of H_2 was determined in the temperature range of 323–533 K of the profile.

H_2–D_2 isotope exchange was carried out using a plug flow quartz reactor at ambient pressure with an online quadrupole mass spectrometer (ThermoStar-GSD 320) following the earlier literature.[3] Typically, 20 mg of catalyst was pre-reduced under H_2 flow at 673 K for 2 h and purged with Ar flow for 2 h at the same temperature. After cooling down to 373 K, a mixture of H_2 (10 kPa) and D_2 (10 kPa) was injected into the reactor.

UV-Vis-NIR spectra of the materials were taken using a Shimadzu optical spectrometer in reflectance mode. The bandgap of material was determined by Tauc plot and baseline method proposed by Makuła et al.[4]

$$\left(\frac{(1-R)^2}{2R}h\nu\right)^n = A(h\nu - E_g), \quad (2)$$

where R is reflectance, $h\nu$ is the photon energy, A is a proportionality constant, and E_g is the bandgap. The exponent n denotes the nature of transition. The values for n (0.5 for metal oxides, and 3 for carbons) were adopted according to the literature.[4,5]

X-ray photoelectron spectroscopy (XPS) were carried out using a Thermo Scientific ESCALAB250 with a Al Kα monochromatic X-ray source ($h\nu$ = 1486.6 eV, 9 mA, 15 kV). The gas treatments were carried out using a lab-made *in situ* cell and gas flowing system to prevent air exposure. Typically, the spectra ware measured after the given gas treatment and subsequent cooling to room temperature, He purging, and evacuation. The spectra denoted as pristine were measured after degassing using He flow at 673 K for 2 h. Next, samples were treated by H_2 at 673 K for 2 h. After cooling down to room temperature, the *in situ* cell was purged by He for 1 h to desorb weakly bound adsorbates. The sample was introduced into the XPS chamber without any air exposure and spectra were recorded at room temperature under the ultrahigh vacuum condition. The sequential spectra measurement after CO_2 (at 533 K) and CO_2+H_2 (1:4 ratio, at 533 K) treatment were carried out similarly. The peak deconvolution was conducted by XPSpeak41 software. Baselines were made by a Shirley type background curve. To deconvolute Zn $2p_{3/2}$ spectra, full width at half maximum (FWHM) and Gaussian-Lorentzian% of peaks were fixed as 2.5–3.1 eV and 25–50%, respectively. The portion of Zn species in Zn $2p_{3/2}$ were determined by their area. Zr 3d spectra were first deconvoluted into a pair of Zr $3d_{5/2}$ and Zr $3d_{3/2}$ doublet with a fixed area ratio of Zr $3d_{5/2}$ over Zr $3d_{3/2}$ of 1.50. Further deconvolutions were carried out with fixed FWHM (1.35–1.60 eV) and Gaussian-Lorentzian% (10–30%) of each peak.

X-ray absorption spectroscopy (**XAS**) was conducted at beamline 01C1 at the National Synchrotron Radiation Research Center, Taiwan. The fresh $ZnZrO_x$ and Pd/CNT+$ZnZrO_x$ catalysts were transferred to *in situ* cell and pretreated with H_2 at 673 K for 2 h like the pretreatment of the reaction condition. For the used $ZnZrO_x$ and Pd/CNT+$ZnZrO_x$ catalysts, the samples recovered from the reactor were immediately transferred to a glass tube and sealed using a PTFE cap equipped with a Viton o-ring to minimize air exposure. Prior to XAS measurement, the used catalysts were transferred to *in situ* cell and pretreated with N_2 at 673 K for 2 h to degas any impurities adsorbed onto catalyst during sample transfer. Here, N_2 was used instead of H_2 to avoid any artificial reduction of the catalyst component. After cooling down the cell to room temperature, the Zn K-edge and Zr K-edge spectra were recorded in the transmission mode. The reference spectra of Zn foil, Zr foil, ZnO, and ZrO_2 were collected together for the comparison. The XAS spectra were analyzed using the Athena and Artemis software included in

the Demeter package. The Fourier transformation of the k^3-weighted extended X-ray absorption fine structure (EXAFS) from k space to R space was carried out over the k range 2.0–12.6 Å^{-1}. A part of the Fourier-transformed EXAFS in the R range of 1.0–2.8 Å were inversely Fourier-transformed, followed by the analysis with a curve fitting method in the k range of 3.0–12.0 Å^{-1}. The back-scattering amplitude and phase shift parameters were simulated with FEFF 6L and used to perform the curve fitting procedure. The amplitude reduction factors (S_o^2) of Zn and Zr were determined by fitting the spectra of reference Zn foil (S_o^2: 1.10) and Zr foil (S_o^2: 0.965), respectively.

Catalytic test and reaction kinetics study

CO_2 hydrogenation under various conditions were carried out using an automatic multi-channel high-pressure flow reactor. The detailed calculation procedure is represented as below.

First, the volume-change-factor (V.C.F.) was calculated by using internal standard Ar as follows:

$$\text{V.C.F.} = \frac{A_{Ar,inlet}}{A_{Ar}}, \quad (3)$$

where $A_{\text{Ar,inlet}}$ and A_{Ar} are the peak area of Ar in inlet and outlet, respectively.

The molar flow rate of CO_2 in inlet ($n_{\text{CO2,inlet}}$) was calculated as:

$$n_{\text{CO2,inlet}}\ (\text{mmol min}^{-1}) = (\text{CO}_2\%\ \text{in inlet} \times 0.01) \times \text{molar reactant flow rate}, \quad (4)$$

where molar reactant flow rate is determined by flow rate (cm^3 min^{-1}, STP) and gas constant (R).

The molar flow rate of species i in outlet (n_i) was calculated as follows:

$$n_i\ (\text{mmol min}^{-1}) = (\text{mol\% of species } i \text{ in outlet} \times 0.01) \times (\text{molar reactant flow rate}) \times \text{V.C.F.}, \quad (5)$$

where the mol% of species i in outlet is determined by GC area of species i with prepared calibration curve. Thermal conductivity detector (TCD) was used for CO_2 and CO, while flame ionization detector (FID) was used for methanol, and etc.

CO_2 conversion, methanol (MeOH) selectivity, and space time yield of methanol (STY_{MeOH}) were calculated with the following equations:

$$\text{CO}_2\ \text{conversion\%} = \frac{n_{\text{CO2,inlet}} - n_{\text{CO2}}}{n_{\text{CO2,inlet}}} \times 100\ \%, \quad (6)$$

$$\text{MeOH selectivity\%} = \frac{n_{MeOH}}{\Sigma\, n_{product}} \times 100\ \%, \quad (7)$$

$$\text{STY}_{\text{MeOH}}\ (\text{g g}_{\text{cat.}}^{-1}\ \text{h}^{-1}) = \frac{GHSV\ (cm^3_{STP}\ g_{cat.}{}^{-1} h^{-1})}{22.4\ (cm^3_{STP}\ mmol^{-1})} \times (\text{CO}_2\%\ in\ inlet \times 0.01) \times (\text{CO}_2\ conversion\% \times 0.01) \times (\text{MeOH}\ selectivity\% \times 0.01) \times Mw_{\text{MeOH}}\ (\text{g mol}^{-1}) \times 0.001\ (\text{mol mmol}^{-1}), \quad (8)$$

where Mw_{MeOH} is the molecular weight of methanol (32 g mol^{-1}).

During the long-term test, cumulative methanol production (Q_{MeOH}) over the catalyst was calculated by integrating the STY_{MeOH} over the time-on-stream.

Q_{MeOH} (g $g_{cat.}^{-1}$) = $\int_0^t STY_{MeOH}(t)\,dt$, (9)

where t is time-on-stream (h).

The reaction kinetic study was conducted in low CO_2 conversion regimes (see Section 3 in Supplementary Information) to avoid any mass transfer and thermodynamic limitations. Typical GHSV condition and the resultant CO_2 conversion for each catalyst are represented in Supplementary Table 2. The methanol formation rate (r_{MeOH}) was calculated by using the equation shown below.

$$r_{MeOH}\ (\text{mmol } g_{ZnZrOx}^{-1}\ h^{-1}) = \frac{GHSV\ (cm^3_{STP}\ g_{cat.}^{-1} h^{-1})}{22.4\ (cm^3_{STP}\ mmol^{-1})} \times (CO_2\%\ in\ inlet \times 0.01) \times (CO_2\ conversion\% \times 0.01) \times (\text{MeOH}\ selectivity\% \times 0.01) \times \text{normalizing ratio}\ \left(\frac{g_{cat.}}{g_{ZnZrOx}}\right). \quad (10)$$

Notably, r_{MeOH} is represented with respect to the mass of $ZnZrO_x$. Typically, the normalizing ratio is 1.5 for mixture catalysts (Pd/*support*:$ZnZrO_x$ = 1:2 mass ratio), 1.01 for Pd/$ZnZrO_x$, and 1.0 for standalone $ZnZrO_x$.

The reaction order of CO_2 was analyzed at 533 K and 5 MPa by controlling the partial pressure of CO_2 ($P(CO_2)$) from 0.5 to 0.95 MPa under a fixed partial pressure of H_2 ($P(H_2)$) at 3.8 MPa, and the reaction order of H_2 was analysed similarly while controlling $P(H_2)$ from 2.6 to 4.1 MPa under a fixed $P(CO_2)$ at 0.65 MPa. Ar was used as a balance gas. To calculate the apparent activation energy (E_a) using the Arrhenius equation, the data were acquired at 493–553 K, 5 MPa, CO_2/H2/Ar = 19/76/5.

Since carbon imbalance leads to a huge error on CO_2 conversion%, the carbon balance was monitored during the reaction to check the validity of the calculation method.

$$\text{Carbon balance\%} = \frac{\sum n_{product}}{n_{CO2,inlet} - n_{CO2}} \times 100\ \%. \quad (11)$$

Typically, the carbon balance% was 99.1–99.9% during the reaction kinetic analysis and 97.9–101.1% during the long-term test.

Details of computational studies

Oxide slabs were constructed from the stable (101) plane[6,7] of tetragonal ZrO_2 bulk using the experimental lattice parameters $a = b = 3.612$ Å, $c = 5.212$ Å[6,7] and contained 3 layers (4 O and 2 Zr per layer, thickness of about 0.69 nm) with the bottom layer fixed in the experimental bulk positions. To obtain the experimentally suggested Zn:Zr ratio of 1:4 (Supplementary Table 1), the 1×1 ZrO_2(101) surface cell (with dimensions of surface lattice vectors of 6.34 Å by 3.61 Å) was transformed, using the transformation matrix [(1,2),(-2,1)], into a supercell containing 20 O and 10 Zr atoms per layer (length of lattice vectors: 9.60 Å by 13.18 Å), and two surface Zr and two O atoms were substituted by Zn atoms, thus, obtaining a $ZnZrO_x$(101) supercell. We also removed two surface O atoms from these models to maintain charge balance in the oxide. Based on these criteria, we constructed different models with Zn–Zn distance of 7.296 Å or 6.275 Å, and the model with the lowest relative energy was chosen for further studies (Supplementary Fig. 21 and 22). Energies of O vacancy formation were calculated as:

$$E_{O\ vac} = E[O\ vac/cat] - E[cat] + E[H_2O] - E[H_2], \quad (12)$$

where E[O vac/cat] is the total DFT energy of the slab with surface O vacancy, while E[cat], E[H_2O], and E[H_2] are the total DFT energies of the unmodified slab and gas-phase H_2O and H_2 molecules.

To consider O vacancies for hydrogenated surface in which 1 H atom adsorbed on O and another on Zn, all the possible position of O atom from the surface and subsurface or an OH species vicinal to H–Zn was removed; in the latter case, the vacancy formation energy is

$$E_{OH\ vac} = E[OH\ vac/cat] – E[cat] + E[H_2O] – 0.5\ E[H_2], \quad (13)$$

where E[OH vac/cat] is the total DFT energy of the surface with the OH pair removed.

For the bare $ZnZrO_x$, the most stable model contained 2 O vacancies and a third O vacancy led to unstable structures (Supplementary Fig. 23). After hydrogenating this model, we found that further reduction of the oxide is still unfeasible due to high formation energies of the third vacancy ranging from 1.4 eV to 3.8 eV (Supplementary Fig. 24). Based on this analysis, the $Zn_2Zr_{n-2}O_{2n-2}$ supercell containing 2 O vacancies was employed for further calculations as the most thermodynamically stable surface composition of the catalyst under reaction conditions where n is the total number of Zr atoms.

The adsorption energies of H were calculated as

$$G_{ads.} = G[H/cat] – G[cat] – 0.5\ G[H_2], \quad (14)$$

where, G[H/cat] is the Gibbs total energy of the adsorbed system and G[cat] = E[cat] is the total energy of the isolated catalyst model.

The O-Zn pair was identified as the most stable adsorption sites for H binding on the oxide (Supplementary Fig. 25).

Graphene and CNT were built using C=C distance of 1.42 Å. We tested three CNT with diameters close to 7 Å; namely, the armchair CNT(5,5), zigzag CNT(9,0) and CNT(8,0), with calculated optical band gaps of 0.07 eV, 0.14 eV and 0.58 eV, respectively. These CNT showed adsorption energies of H varying only within 0.08 eV and our calculations using CNT(5,5) and CNT(9,0) led to the same conclusions for H adsorption on CNT/$ZnZrO_x$ and H movement from CNT to $ZnZrO_x$. Therefore, the discussion in the main article focuses on CNT(5,5) for the sake of brevity.

Models of Pd nanoparticles supported on graphene were obtained from Wulff-constructed Pd nanoparticles truncated from the [8] direction with 1.56 nm diameter of the widest Pd square along {001} and 1.20 nm height (aspect ratio of 1.3) and a diameter of the Pd square at the interface of 1.16 nm, which is similar to nanoparticles observed by electron microscopic images in previous experimental studies.[9,10] Namely, our studies considered Pd_{127} nanoparticle with a (100) facet in contact with a 10×10 graphene supercell. We screened all possible high-symmetry structures of the Pd/graphene interface by aligning the center of mass of the nanoparticle to each of the high-symmetry sites of graphene and screening various rotation angles (0°, 15°, 30°, 45°, 60°, 75°, 90°) of the Pd nanoparticle around the axis normal to graphene passing through the center of mass of Pd (Supplementary Fig. 26).

The model containing CNTs in contact with $ZnZrO_x$ surface was constructed by constructing CNT and $ZnZrO_x$

supercells with closely matching lattice parameters using in-house lattice matching algorithm. In particular, CNT(5,5)/ZnZrO_x(101) model was constructed by locating (4×1) CNT(5,5) supercell compressed by 2.33% to fit with the (1×2) ZnZrO_x(101) supercell (9.61 Å by 26.37 Å combined cell) based on the aforementioned lattice parameters derived from experimental values. The compression has negligible impact in the properties of the freestanding CNT, for instance, destabilizing it by only ~20 meV/atom and without inducing qualitative change in the C local density of states. The CNT was positioned to have one C atom exactly above the strongest H-binding O site of the ZnZrO_x. Adsorption of a single H atom was studied on the external wall of CNT, because the adsorption of one H on the interior wall is much weaker (Gibbs adsorption energy of 2.28 eV) due to the formation of unstable distorted walls.[11] For the fully hydrogenated CNT(5,5), the H atoms alternate binding on the inner and outer walls, and the length of the lattice vector along the tube length for the unit cell (20 C and 20 H atoms) was expanded from the 2.46 Å to 2.55 Å to minimize the energy of the system. The strain from lattice mismatch with the oxide was minimized to −1.57 % by combining 5 unit cells of the hydrogenated CNT with the ZnZrO_x(101) supercell scaled by the [(1,1),(0,2)] transformation matrix.

2. Supplementary tables and figures

Supplementary Table 1. Structural characterization of catalysts

**Catalyst**	**Surface Zn/(Zn+Zr)%**[a]	S_{BET}[b] **(m^2 g^{-1})**	**Pd wt% in total catalyst**	**Pd:ZnZrO$_x$ mass ratio**	D_{Pd}[c] **(%)**
ZnZrO$_x$	20.8	42	none	none	n.d.[d] (n.d.[d])
Pd/ZnZrO$_x$	n.a.[e]	50	1	1:99	18 (12)
Pd/CNT	none	101	2	-	21 (20)
Pd/CNT+ZnZrO$_x$	n.a.[e]	-	0.67	1:100	20 (23)
CNT+ZnZrO$_x$	20.6[f]	-	none	none	none
Pd/GNP	none	711	2	-	16
Pd/GNP+ZnZrO$_x$	n.a.[e]	-	0.67	1:100	16
Pd/AC	none	539	2	-	23
Pd/AC+ZnZrO$_x$	n.a.[e]	-	0.67	1:100	22
Pd/SiO_2	none	392	2	-	18 (19)
Pd/SiO_2+ZnZrO$_x$	n.a.[e]	-	0.67	1:100	18
Pd/TiO_2	none	52	2	-	22 (20)
Pd/TiO_2+ZnZrO$_x$	n.a.[e]	-	0.67	1:100	20

[a] Measured by XPS.
[b] BET surface area
[c] Dispersion of Pd (D_{Pd}) determined by H_2-chemisorption at 343 K. The values in parenthesis indicate the value obtained from CO-chemisorption at 343 K.
[d] Cannot be determined. The chemisorption amount was measured as almost 0.
[e] Not available due to the distortion of Zr signal in the presence of Pd.
[f] The value indicates physical mixing does not affect surface Zn/(Zn+Zr).

Supplementary Table 2. GHSV condition and CO_2 hydrogenation result for each catalyst shown in Fig. 2a. The average values and standard deviations were determined by at least three measurements (reaction condition: 533 K, 5 MPa, $CO_2/H_2/Ar$=19/76/5).

**Catalyst**	**GHSV (cm^3_{STP} $g_{cat.}^{-1}$ h^{-1})**	**CO_2 conv.%**	**MeOH sel.%**	**CO sel.%**	**CH_4 sel.%**	**DME sel.%**
$ZnZrO_x$	24000	0.94±0.09	88±1	12±1	n.d.[a]	n.d.[a]
Pd/$ZnZrO_x$	48000	1.20±0.07	80±2	20±2	n.d.[a]	n.d.[a]
Pd/CNT+$ZnZrO_x$	144000	0.99±0.04	75±2	25±2	n.d.[a]	n.d.[a]
Pd/$ZnZrO_x$+CNT	90000	1.36±0.06	49±2	51±2	n.d.[a]	n.d.[a]
CNT+$ZnZrO_x$	24000	0.64±0.10	89±1	11±1	n.d.[a]	n.d.[a]
Pd/GNP+$ZnZrO_x$	72000	0.96±0.06	80±2	20±2	n.d.[a]	n.d.[a]
Pd/AC+$ZnZrO_x$	72000	1.20±0.06	78±3	78±3	n.d.[a]	n.d.[a]
Pd/SiO_2+$ZnZrO_x$	24000	1.25±0.18	54±4	46±4	0.11±0.04	n.d.[a]
Pd/TiO_2+$ZnZrO_x$	96000	1.08±0.14	18±1	78±1	3.40±0.06	0.14±0.01

[a] Not detected.

Supplementary Table 3. CO_2 hydrogenation results for $ZnZrO_x$, $Pd/ZnZrO_x$, and $Pd/CNT+ZnZrO_x$ catalysts. (reaction condition: 5 MPa, $CO_2/H_2/Ar$=19/76/5, GHSV = 24000 cm^3_{STP} $g_{cat.}^{-1}$ h^{-1}).

**Catalyst**	**Reaction temp. (K)**	**CO_2 conv.%**	**MeOH sel.%**	**CO sel.%**	**CH_4 sel.%**	**DME sel.%**
$ZnZrO_x$	513	0.28	100	n.d.[a]	n.d.[a]	n.d.[a]
	533	0.87	87.1	12.9	n.d.[a]	n.d.[a]
	553	2.17	83.7	16.3	n.d.[a]	n.d.[a]
	573	4.39	85.1	14.9	n.d.[a]	n.d.[a]
	593	7.69	83.0	16.3	n.d.[a]	0.67
	613	11.6	77.3	21.7	n.d.[a]	1.00
	633	15.4	65.0	33.5	0.13	1.30
$Pd/ZnZrO_x$	513	0.73	88.1	11.9	n.d.[a]	n.d.[a]
	533	2.00	81.7	18.3	n.d.[a]	n.d.[a]
	553	4.09	82.9	17.1	n.d.[a]	n.d.[a]
	573	7.32	83.9	16.1	n.d.[a]	n.d.[a]
	593	11.6	81.5	18.5	0.02	n.d.[a]
	613	15.4	73.7	25.8	0.21	0.29
	633	17.8	58.1	41.1	0.30	0.41
$Pd/CNT+ZnZrO_x$	513	3.13	81.8	18.2	n.d.[a]	n.d.[a]
	533	4.76	79.8	20.2	n.d.[a]	n.d.[a]
	553	7.37	76.2	23.8	n.d.[a]	n.d.[a]
	573	11.7	72.1	27.9	n.d.[a]	n.d.[a]
	593	18.1	66.3	33.7	0.06	n.d.[a]
	613	22.3	52.3	47.4	0.19	0.20

[a] Not detected.

Supplementary Table 4. Summary of the catalytic performances of CO_2 hydrogenation to methanol over $ZnZrO_x$ and metal-promoted $ZnZrO_x$ catalysts.

**Catalyst**	**Reaction condition**	**STY_{MeOH} ($g\ g_{cat.}^{-1}\ h^{-1}$)[a,b]**	**Ref.**
$ZnZrO_x$ (Coprecipitation)	320 °C, 5.0 MPa, H_2/CO_2=3, 24000 $cm^3{}_{STP}\ g_{cat.}^{-1}\ h^{-1}$	0.730	6
	315 °C, 5.0 MPa, H_2/CO_2=4, 24000 $cm^3{}_{STP}\ g_{cat.}^{-1}\ h^{-1}$	0.593	
$ZnZrO_x$ (EISA process)	320 °C, 5.5 MPa, H_2/CO_2=3, 24000 $cm^3{}_{STP}\ g_{cat.}^{-1}\ h^{-1}$	0.707	12
$ZnZrO_x$ (Aerogel synthesis)	340 °C, 4.0 MPa, H_2/CO_2=3, 21180 $cm^3{}_{STP}\ g_{cat.}^{-1}\ h^{-1}$	0.547	13
$ZnZrO_x$ (Coprecipitation with micro-reactor)	320 °C, 3.0 MPa, H_2/CO_2=3, 12000 $cm^3{}_{STP}\ g_{cat.}^{-1}\ h^{-1}$	0.350	14
0.8 at.% Pd-$ZnZrO_x$ (coprecipitation)	320 °C, 5.0 MPa, H_2/CO_2=4, 24000 $cm^3{}_{STP}\ g_{cat.}^{-1}\ h^{-1}$	0.630 (0.710)[c]	15
0.1 wt% Pd-$ZnZrO_x$ (coprecipitation)	320 °C, 5.0 MPa, H_2/CO_2=3, 30000 $cm^3{}_{STP}\ g_{cat.}^{-1}\ h^{-1}$	0.735	16
0.5 at.% Cu-$ZnZrO_x$ (coprecipitation)	310 °C, 4.5 MPa, H_2/CO_2=3, 10800 $cm^3{}_{STP}\ g_{cat.}^{-1}\ h^{-1}$	0.300	17
0.02 at.% Pd-$ZnZrO_x$ (coprecipitation)	320 °C, 4.5 MPa, H_2/CO_2=3, 10800 $cm^3{}_{STP}\ g_{cat.}^{-1}\ h^{-1}$	0.303	
0.02 at.% Pt-$ZnZrO_x$ (coprecipitation)		0.290	
$ZnZrO_x$	320 °C, 5.0 MPa, H_2/CO_2=4, 24000 $cm^3{}_{STP}\ g_{cat.}^{-1}\ h^{-1}$	0.430	This work
Pd/CNT+$ZnZrO_x$ (physical mixing)		0.900[d]	

[a] If it is not shown in the literature, the value was estimated by using CO_2 conversion, methanol selectivity, space velocity, and concentration of CO_2 in the reactant gas mixture.
[b] As some literature reported dimethyl ether (DME) as the secondary product from methanol (MeOH) condensation, the DME production rate was also included under the assumption '1 DME = 2 MeOH'.
[c] The value in parenthesis indicates the STY_{MeOH} after 100 h reaction.
[d] The value in parenthesis indicates the STY_{MeOH} after 600 h reaction.

Supplementary Table 5. Comparison of state-of-the-art catalysts in CO_2 hydrogenation to methanol.

**Catalysts**	**Reaction conditions**	**TOS (h)**	**STY_{MeOH} ($g\ g_{cat.}^{-1}\ h^{-1}$)**	**Ref.**
$Cu/ZnO/Al_2O_3$[a]	250 °C, 3.0 MPa, H_2/CO_2=3, 30000 $cm^3_{STP}\ g_{cat.}^{-1}\ h^{-1}$	600	0.85	18
$Cu/ZnO/Al_2O_3$	300 °C, 5.0 MPa, H_2/CO_2=4, 20000 $cm^3_{STP}\ g_{cat.}^{-1}\ h^{-1}$	100	0.122	19
$Cu/ZnO/Al_2O_3$[a]	260 °C, 5.0 MPa, H_2/CO_2=4, 24000 $cm^3_{STP}\ g_{cat.}^{-1}\ h^{-1}$	600	0.627	This work
$Cu/ZnO/Al_2O_3$[a]	200 °C, 3.0 MPa, H_2/CO_2=3, 9000 $cm^3_{STP}\ g_{cat.}^{-1}\ h^{-1}$	720	0.119	20
$Cu/ZnO/ZrO_2$	220 °C, 3.0 MPa, H_2/CO_2=3, 6000 $cm^3_{STP}\ g_{cat.}^{-1}\ h^{-1}$	16	0.297	21
Cu/ZnO/5Ga	240 °C, 4.5 MPa, H_2/CO_2=2.8, 18000 $cm^3_{STP}\ g_{cat.}^{-1}\ h^{-1}$	n.s.[b]	0.880	22
$3DOM-Cu/ZnO/ZrO_2$	240 °C, 4.0 MPa, H_2/CO_2=3, 18000 $cm^3_{STP}\ g_{cat.}^{-1}\ h^{-1}$	16	0.747	23
$ZnZrO_x$	320 °C, 5.0 MPa, H_2/CO_2=3, 24000 $cm^3_{STP}\ g_{cat.}^{-1}\ h^{-1}$	500	0.730	6
$ZnZrO_x$	320 °C, 5.0 MPa, H_2/CO_2=4, 24000 $cm^3_{STP}\ g_{cat.}^{-1}\ h^{-1}$	150	0.430	This work
$Pd/CNT+ZnZrO_x$	320 °C, 5.0 MPa, H_2/CO_2=4, 24000 $cm^3_{STP}\ g_{cat.}^{-1}\ h^{-1}$	600	0.900 (1.351)[c]	This work
c-In_2O_3-S	300 °C, 5.0 MPa, H_2/CO_2=4, 9000 $cm^3_{STP}\ g_{cat.}^{-1}\ h^{-1}$	130	0.34	24
In_2O_3/ZrO_2	300 °C, 5.0 MPa, H_2/CO_2=4, 16000 $cm^3_{STP}\ g_{cat.}^{-1}\ h^{-1}$	1000	0.30	19
$CdZrO_x$	300 °C, 2.0 MPa, H_2/CO_2=3, 24000 $cm^3_{STP}\ g_{cat.}^{-1}\ h^{-1}$	3	0.358	25
$GaZnZrO_x$	320 °C, 5.0 MPa, H_2/CO_2=3, 24000 $cm^3_{STP}\ g_{cat.}^{-1}\ h^{-1}$	100	0.630	26
Pd/In_2O_3	280 °C, 5.0 MPa, H_2/CO_2=4, 24000 $cm^3_{STP}\ g_{cat.}^{-1}\ h^{-1}$	90	0.61	27
Pd/In_2O_3	300 °C, 5.0 MPa, H_2/CO_2=4, 21000 $cm^3_{STP}\ g_{cat.}^{-1}\ h^{-1}$	n.s.[b]	0.885	28
Ir/In_2O_3	300 °C, 5.0 MPa, H_2/CO_2=4, 21000 $cm^3_{STP}\ g_{cat.}^{-1}\ h^{-1}$	12	0.75	29
Au/In_2O_3	300 °C, 5.0 MPa, H_2/CO_2=4, 21000 $cm^3_{STP}\ g_{cat.}^{-1}\ h^{-1}$	12	0.47	30
Pt/In_2O_3	300 °C, 4.0 MPa, H_2/CO_2=3, 24000 $cm^3_{STP}\ g_{cat.}^{-1}\ h^{-1}$	n.s.[b]	0.475	31
$InNi_3C_{0.5}$/*m*-ZrO_2	300 °C, 4.0 MPa, H_2/CO_2=3, 24000 $cm^3_{STP}\ g_{cat.}^{-1}\ h^{-1}$	120	0.62	32
$InNi_3C_{0.5}/Fe_3O_4$	320 °C, 5.0 MPa, H_2/CO_2=3, 24000 $cm^3_{STP}\ g_{cat.}^{-1}\ h^{-1}$	500	1.01	32
In@Co	300 °C, 5.0 MPa, H_2/CO_2=4, 27500 $cm^3_{STP}\ g_{cat.}^{-1}\ h^{-1}$	90	0.86	33

[a] Denotes a commercial catalyst.
[b] Not shown in the literature.
[c] Number in parenthesis is the normalized to the active phase ($ZnZrO_x$).

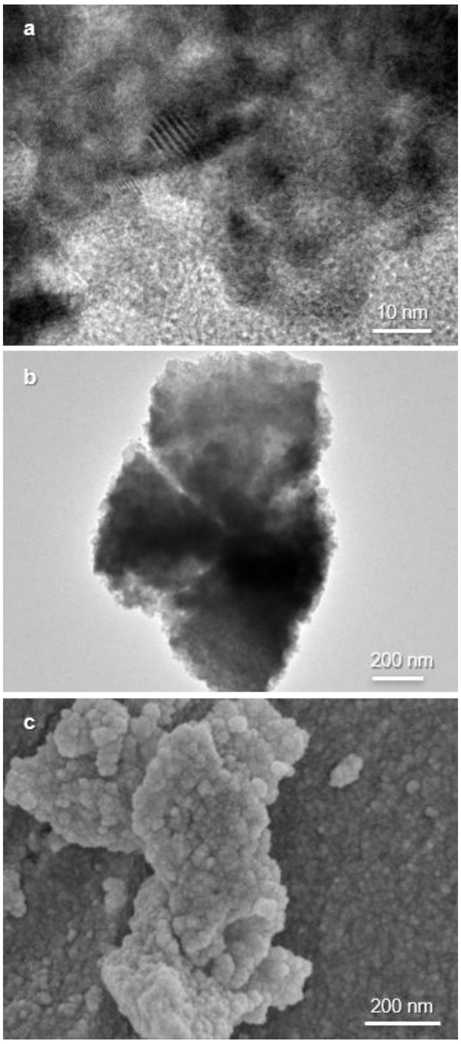

Supplementary Figure 1. a High-magnification TEM image, **b** low-magnification TEM image, and **c** SEM image of $ZnZrO_x$.

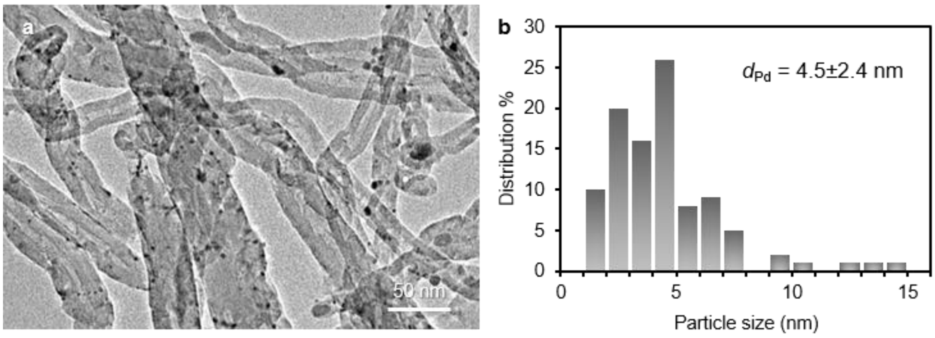

Supplementary Figure 2. a TEM image and **b** Pd particle size distribution of Pd/CNT.

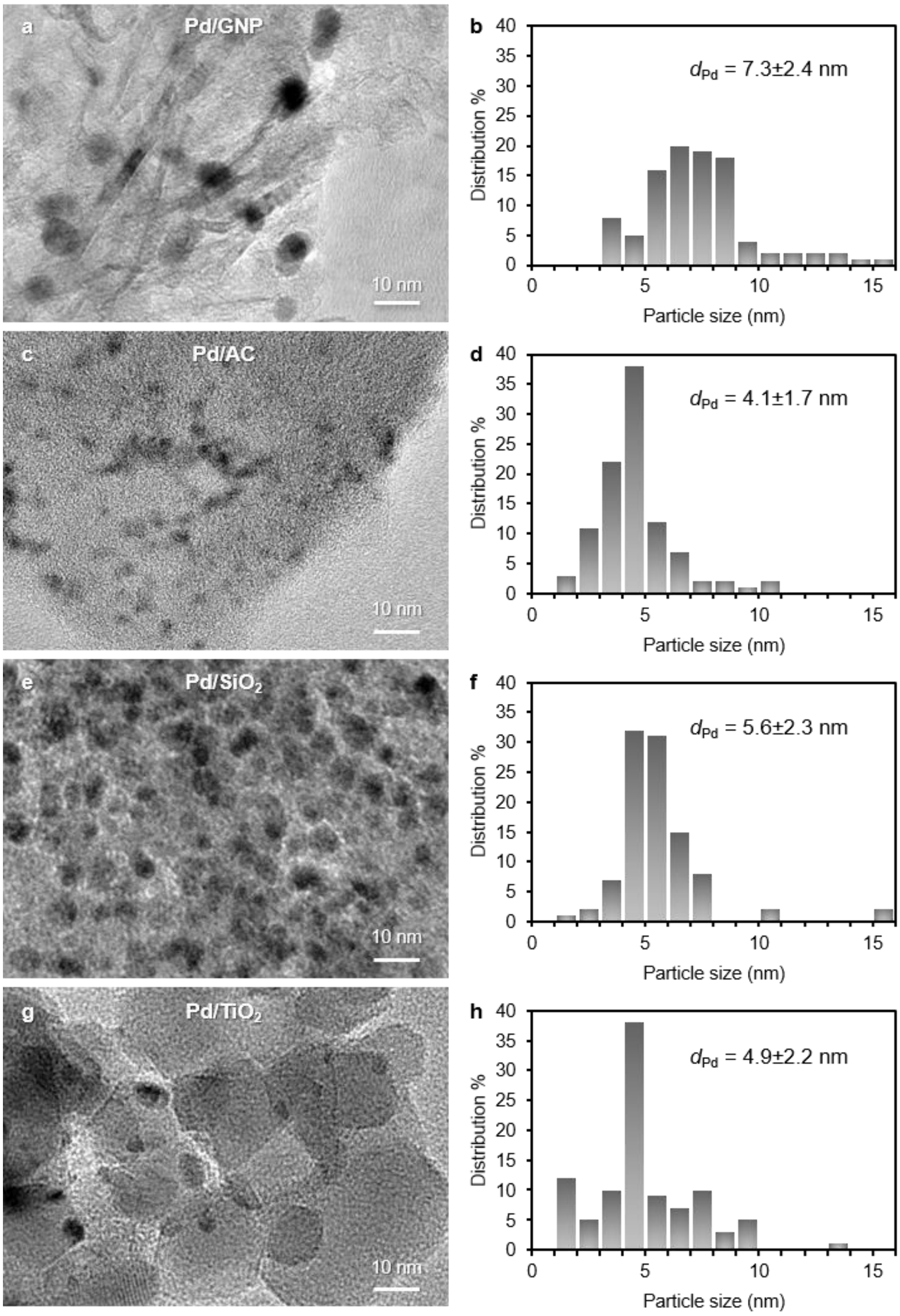

Supplementary Figure 3. (**a, c, e, g**) TEM images and (**b, d, f, h**) Pd particle size distributions of Pd/*support* catalysts. (**a, b**) Pd/GNP, (**c, d**) Pd/AC, (**e, f**) Pd/SiO_2, (**g, h**) Pd/TiO_2.

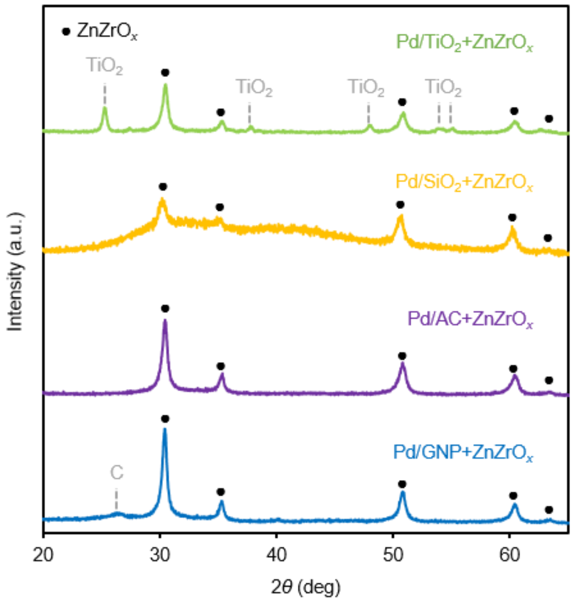

Supplementary Figure 4. Powder XRD patterns of Pd/GNP+ZnZrO$_x$, Pd/AC+ZnZrO$_x$, Pd/SiO$_2$+ZnZrO$_x$, and Pd/TiO$_2$+ZnZrO$_x$.

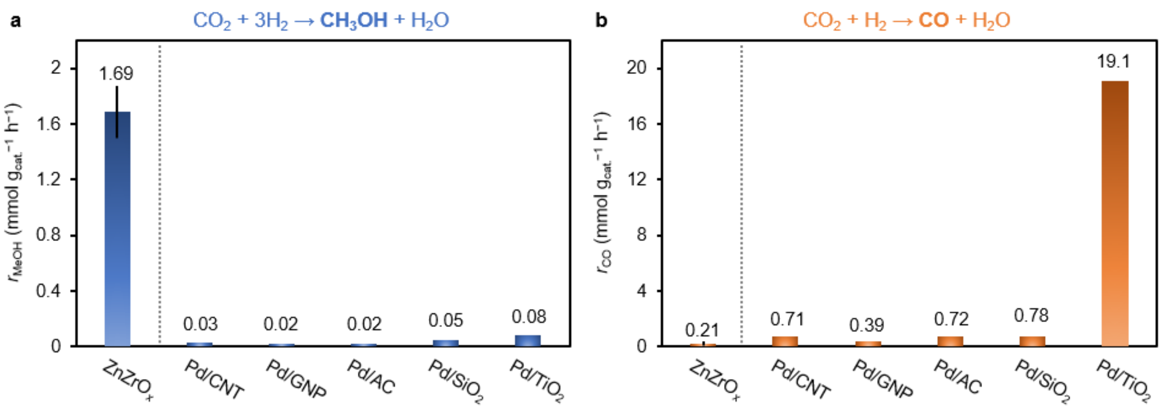

Supplementary Figure 5. a MeOH formation rates, and **b** CO formation rates of standalone Pd/*support* catalysts. The results for ZnZrO$_x$ are included for comparison (533 K, 5 MPa, $CO_2/H_2/Ar=19/76/5$, GHSV 24000 cm^3_{STP} $g_{cat.}^{-1}$ h^{-1} for ZnZrO$_x$, GHSV 48000 cm^3_{STP} $g_{cat.}^{-1}$ h^{-1} for Pd/*support* catalysts).

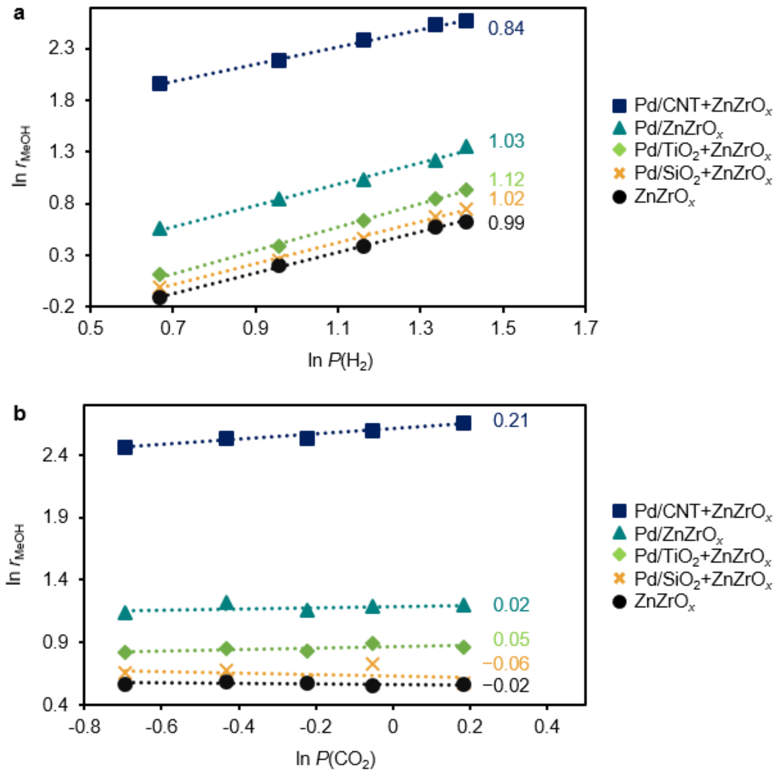

Supplementary Figure 6. Reaction order analysis for **a** H_2, and **b** CO_2 of the catalysts (Condition: 533 K and 5 MPa. GHSVs are shown in Supplementary Table 2). For figure a, $P(H_2)$ were controlled from 2.6 to 4.1 MPa under a fixed $P(CO_2)$ = 0.65 MPa. For figure b, $P(CO_2)$ were controlled from 0.5 to 0.95 MPa under a fixed $P(H_2)$ = 3.8 MPa.

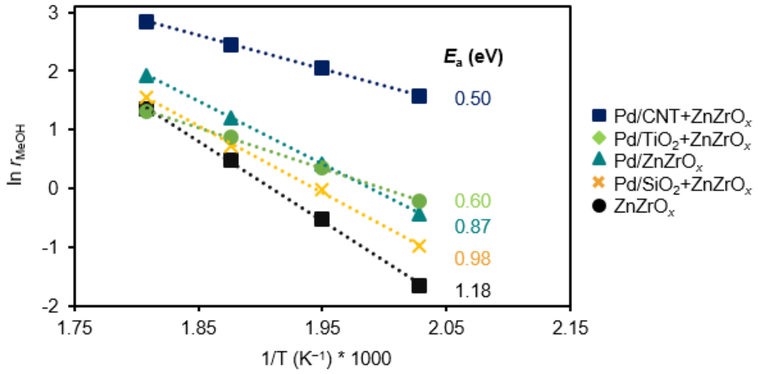

Supplementary Figure 7. Arrhenius plot for determination of the apparent activation energy (E_a) of the catalysts. (Condition: 493–553 K, 5 MPa, and $CO_2/H_2/Ar$ = 19/76/5. GHSVs are shown in Supplementary Table 2)

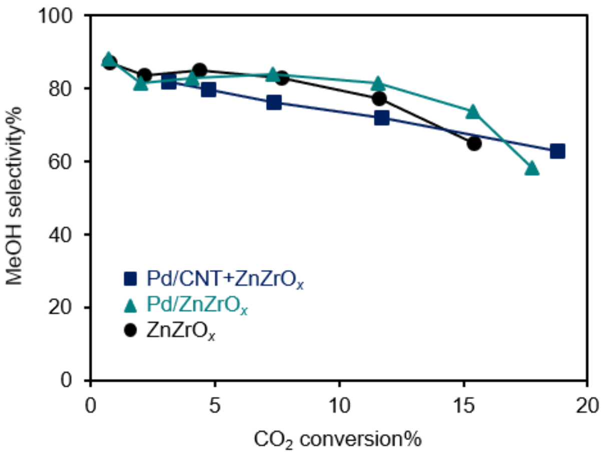

Supplementary Figure 8. Methanol selectivity of ZnZrO$_x$, Pd/ZnZrO$_x$, and Pd/CNT+ZnZrO$_x$ catalysts as a function of CO_2 conversion in the range of 0.7–18% (Condition: 513–633 K, 5 MPa, $CO_2/H_2/Ar$ = 19/76/5, GHSV = 24000 $cm^3\ g_{cat}^{-1}\ h^{-1}$).

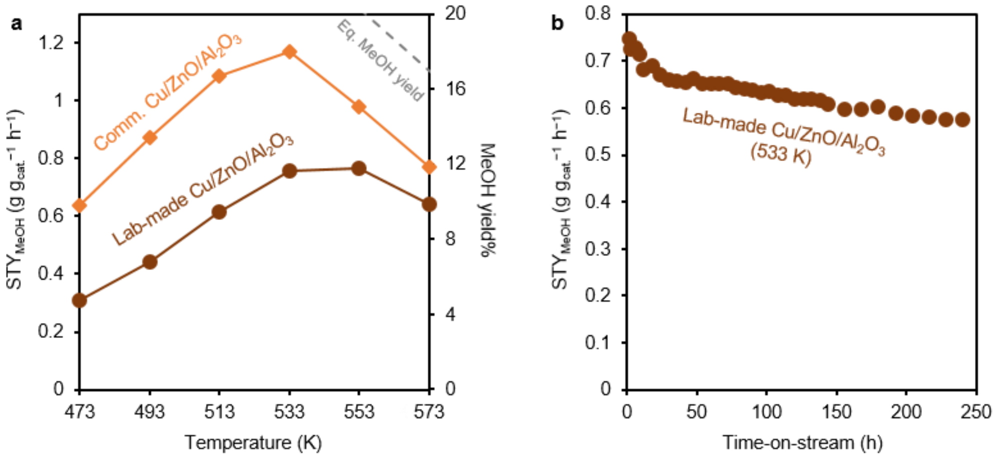

Supplementary Figure 9. Catalytic performance of Cu/ZnO/Al$_2$O$_3$ catalysts. a Methanol yield and STY$_{\mathrm{MeOH}}$ of Cu/ZnO/Al$_2$O$_3$ catalysts as a function of reaction temperature. The dashed line indicates the methanol yield at thermodynamic equilibrium. Cu/ZnO/Al$_2$O$_3$ catalysts commonly show the highest methanol yield at *ca*. 533 K. **b** Long-term catalytic test result of lab-made Cu/ZnO/Al$_2$O$_3$ catalyst at 533 K. The result supports that a rapid deactivation is the general behavior of Cu/ZnO/Al$_2$O$_3$.

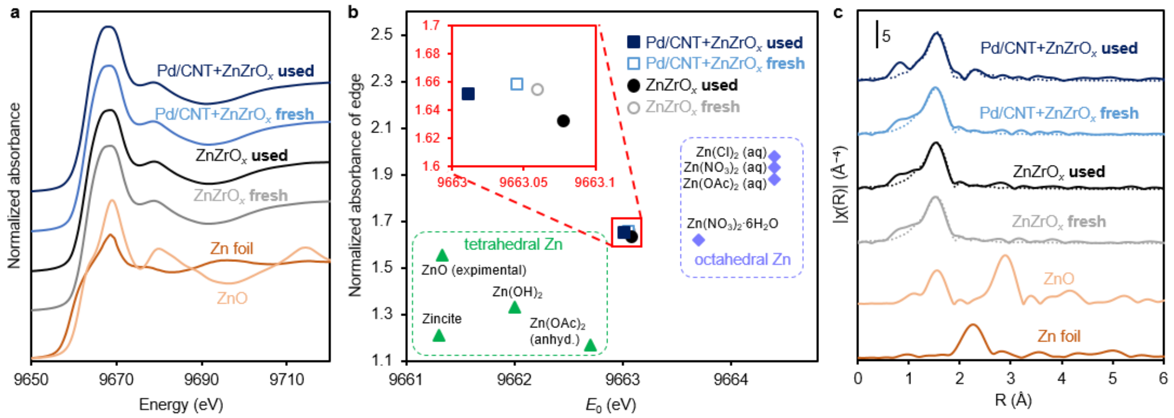

Supplementary Figure 10. Zn K edge XAFS analysis of the catalysts. a Zn *K* edge XANES of the ZnZrO$_x$ and Pd/CNT+ZnZrO$_x$ catalysts before and after reaction. The used catalysts were obtained after the reaction shown in Fig. 3b. **b** Normalized absorbance of edge as a function of E_0 for catalysts as well as standard Zn compounds. Except for ZnO (experimental), the values for standard Zn compounds were adopted from ref.[34]. The result indicates that Zn in ZnZrO$_x$ exists as the intermediate state between tetrahedral and octahedral structure, proving that the solid solution structure of which Zn is highly dispersed in the ZrO$_2$ domain. **c** Zn *K* edge k^3-weighted Fourier transforms of the EXAFS of the ZnZrO$_x$ and Pd/CNT+ZnZrO$_x$ catalysts before and after reaction. The overall results represent that Zn is not changed during the long-term reaction.

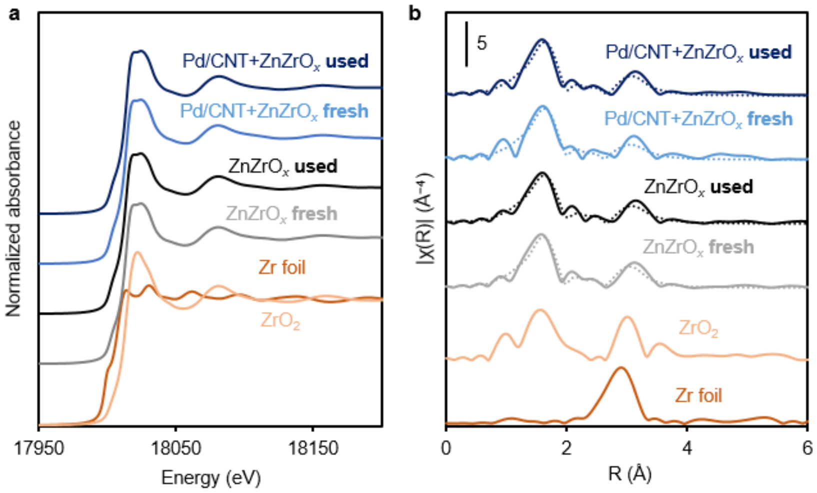

Supplementary Figure 11. Zr K edge XAFS analysis of the catalysts. a Zr *K* edge XANES of the $ZnZrO_x$ and Pd/CNT+$ZnZrO_x$ catalysts before and after reaction. The used catalysts were obtained after the reaction shown in Fig. 3b. **b** Zr *K* edge k^3-weighted Fourier transforms of the EXAFS of the $ZnZrO_x$ and Pd/CNT+$ZnZrO_x$ catalysts before and after reaction. The overall results represent that Zr is not changed during the long-term reaction.

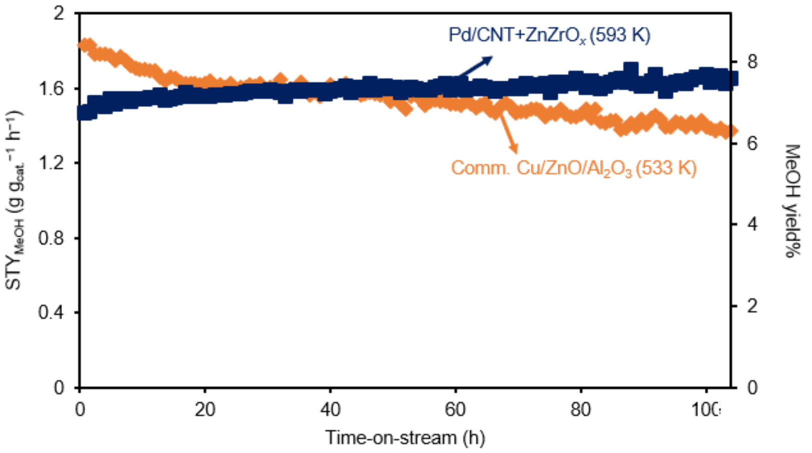

Supplementary Figure 12. Long-term catalytic tests of Pd/CNT+$ZnZrO_x$ at 593 K, and commercial $Cu/ZnO/Al_2O_3$ at 533 K (Condition: 5 MPa, $CO_2/H_2/Ar$ = 19/76/5, GHSV = 80000 cm^3_{STP} $g_{cat.}^{-1}$ h^{-1}). The test was measured far below the thermodynamic equilibrium.

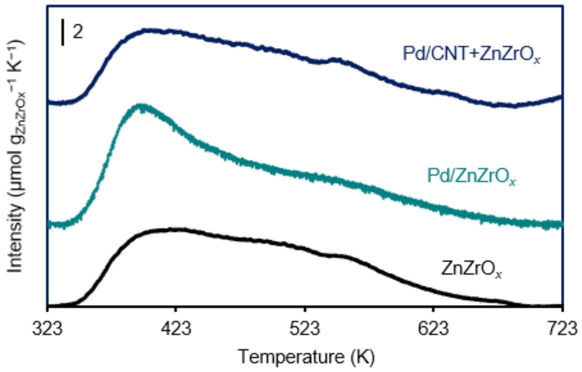

Supplementary Figure 13. CO_2-TPD profiles of $ZnZrO_x$, Pd/$ZnZrO_x$, and Pd/CNT+$ZnZrO_x$. Three catalysts show a similar CO_2 adsorption strength distribution. Pd/$ZnZrO_x$ shows a 21% higher CO_2 adsorption capacity (0.987 mmol g_{ZnZrOx}^{-1}) than $ZnZrO_x$ (0.814 mmol g_{ZnZrOx}^{-1}). This is coherent with their surface area gap (20%, Supplementary Table 1). Pd/CNT+$ZnZrO_x$ shows almost the same adsorption capacity (0.803 mmol g_{ZnZrOx}^{-1}) compared to $ZnZrO_x$,

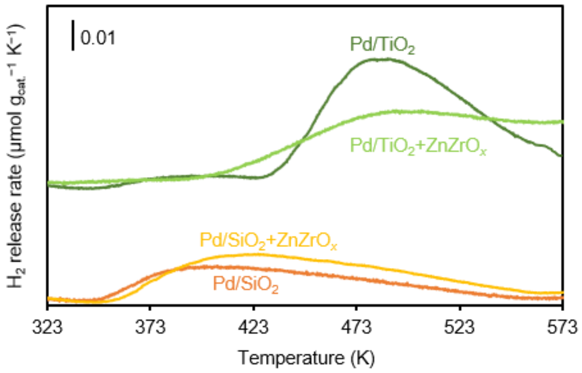

Supplementary Figure 14. H_2-TPD profiles of Pd/SiO_2, Pd/SiO_2+$ZnZrO_x$, Pd/TiO_2, and Pd/TiO_2+$ZnZrO_x$. The amount of reversible H_2 (quantified in the range of 323–533 K) on the catalysts were determined as follows. Pd/SiO_2 = 7.9 μmol $g_{cat.}^{-1}$, Pd/SiO_2+$ZnZrO_x$ = 9.8 μmol $g_{cat.}^{-1}$, Pd/TiO_2 = 20.7 μmol g_{cat}^{-1}, and Pd/TiO_2+$ZnZrO_x$ = 16.4 μmol g_{cat}^{-1}.

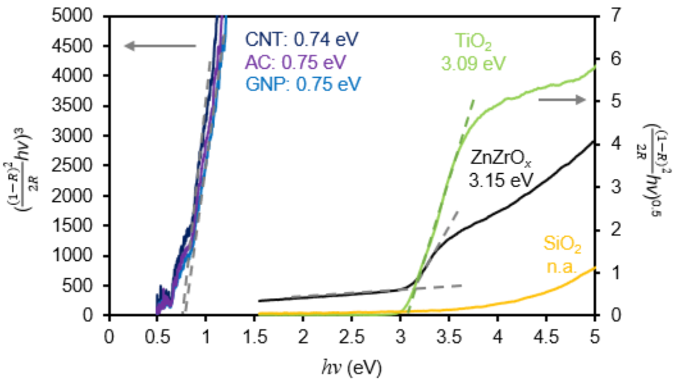

Supplementary Figure 15. Tauc plot of UV-Vis-NIR spectra and bandgap determination for support materials.

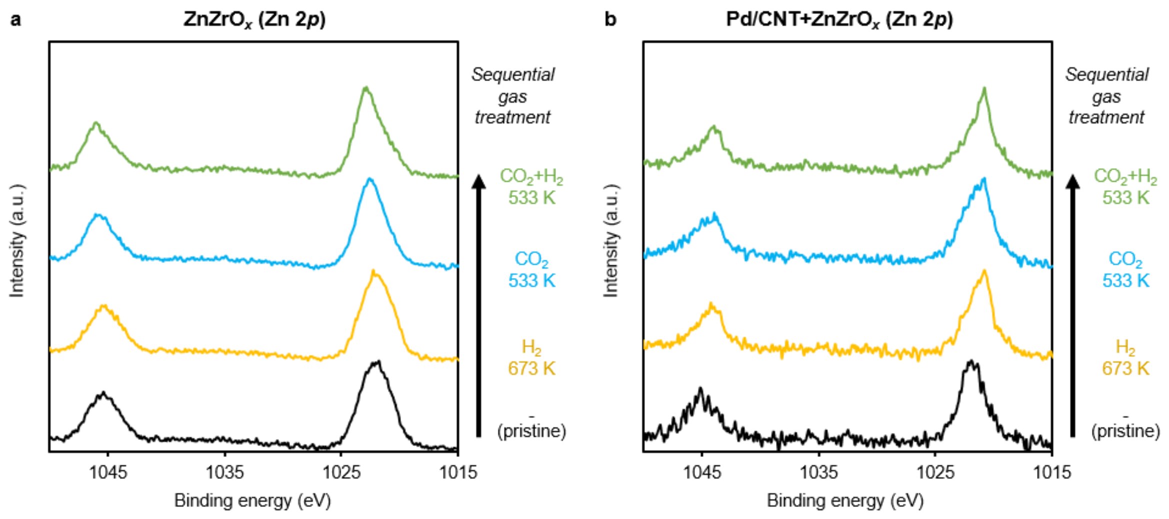

Supplementary Figure 16. Zn 2*p* XPS of **a** ZnZrO$_x$, and **b** Pd/CNT+ZnZrO$_x$ catalysts before and after sequential gas treatments (without sample exposure to air; gas treatment condition: H_2 at 673 K, CO_2 at 533 K, CO_2+H_2 (1:4) at 533 K).

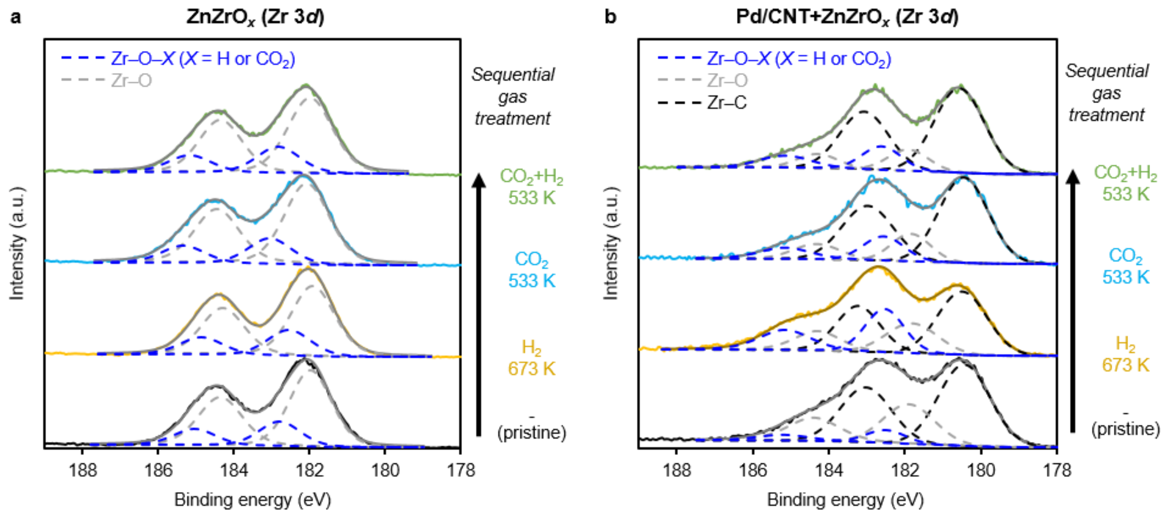

Supplementary Figure 17. Zr 3*d* XPS of **a** ZnZrO$_x$, and **b** Pd/CNT+ZnZrO$_x$ catalysts before and after sequential gas treatments (without sample exposure to air; gas treatment condition: H_2 at 673 K, CO_2 at 533 K, CO_2+H_2 (1:4) at 533 K).

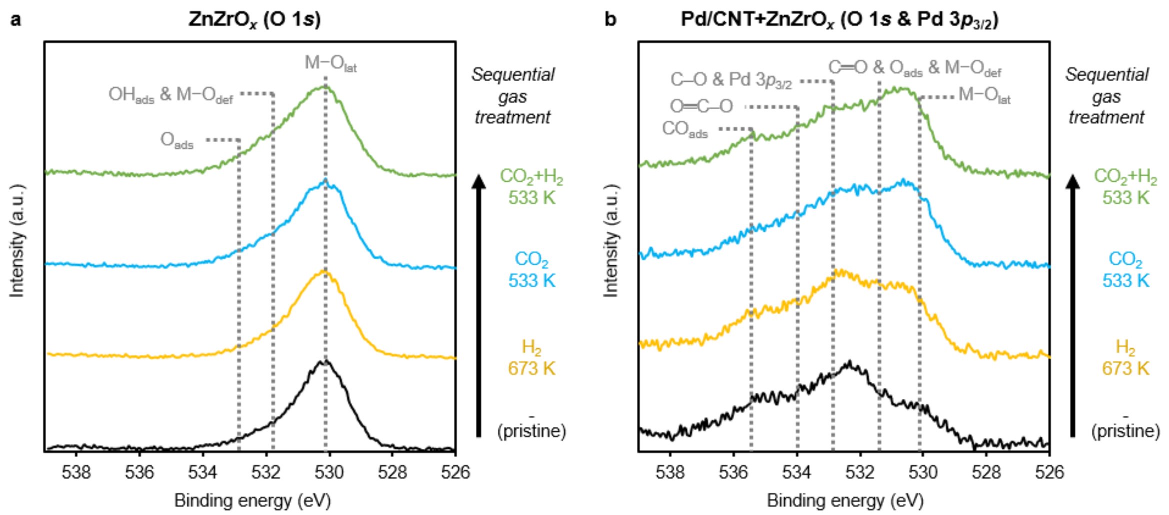

Supplementary Figure 18. O 1*s* XPS of **a** ZnZrO$_x$, and **b** Pd/CNT+ZnZrO$_x$ catalysts before and after sequential gas treatments (without sample exposure to air; gas treatment condition: H_2 at 673 K, CO_2 at 533 K, CO_2+H_2 (1:4) at 533 K).

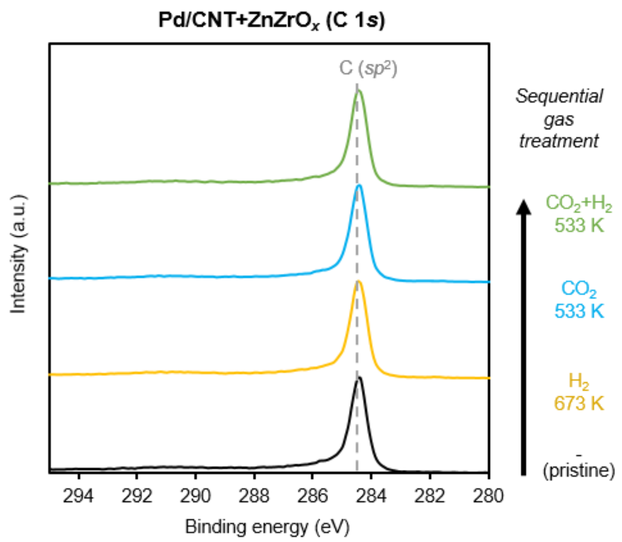

Supplementary Figure 19. C 1*s* XPS of Pd/CNT+ZnZrO$_x$ catalysts before and after sequential gas treatments (without sample exposure to air; gas treatment condition: H_2 at 673 K, CO_2 at 533 K, CO_2+H_2 (1:4) at 533 K).

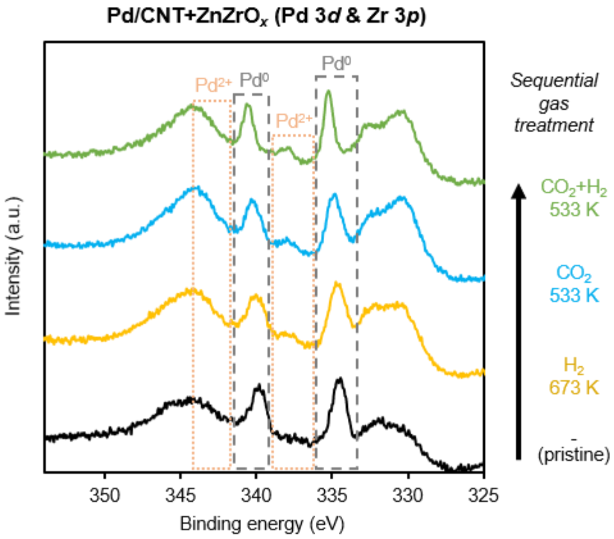

Supplementary Figure 20. Pd 3*d* (and Zr 3*p*) XPS of Pd/CNT+ZnZrO$_x$ catalysts before and after sequential gas treatments (without sample exposure to air; gas treatment condition: H_2 at 673 K, CO_2 at 533 K, CO_2+H_2 (1:4) at 533 K).

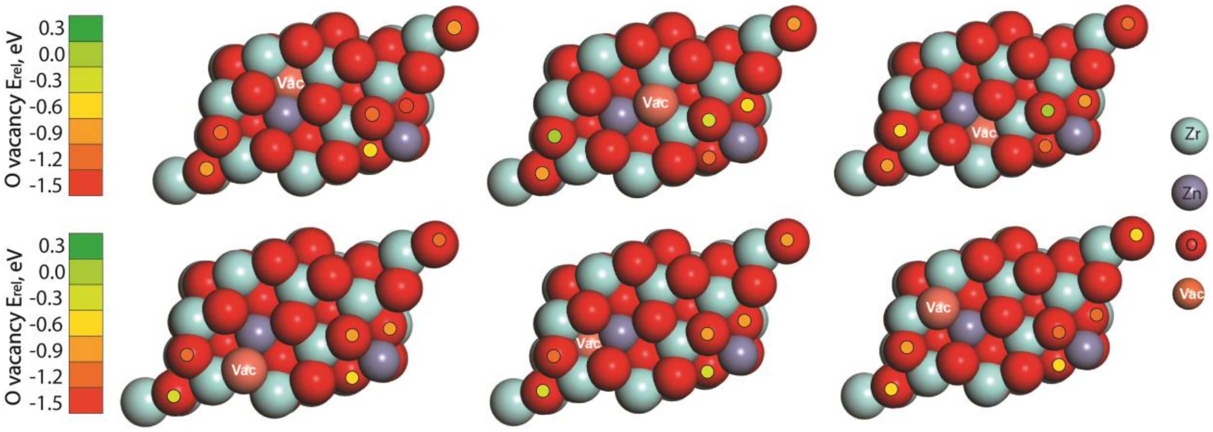

Supplementary Figure 21. Relative DFT energies (E_{rel}) of $Zn_2Zr_{n-2}O_{2n-2}$ structure with two O vacancies per unit cell of a model with Zn–Zn distance of 7.296 Å, formed via substitution of two Zn atoms to Zr to acquire the Zn to Zr concentration on surface in 1:4 ratio. Second O vacancy is generated near Zn atom with respect to first O vacancy highlighted as “Vac” positions in these structures. Note that O vacancies are created near Zn atoms.

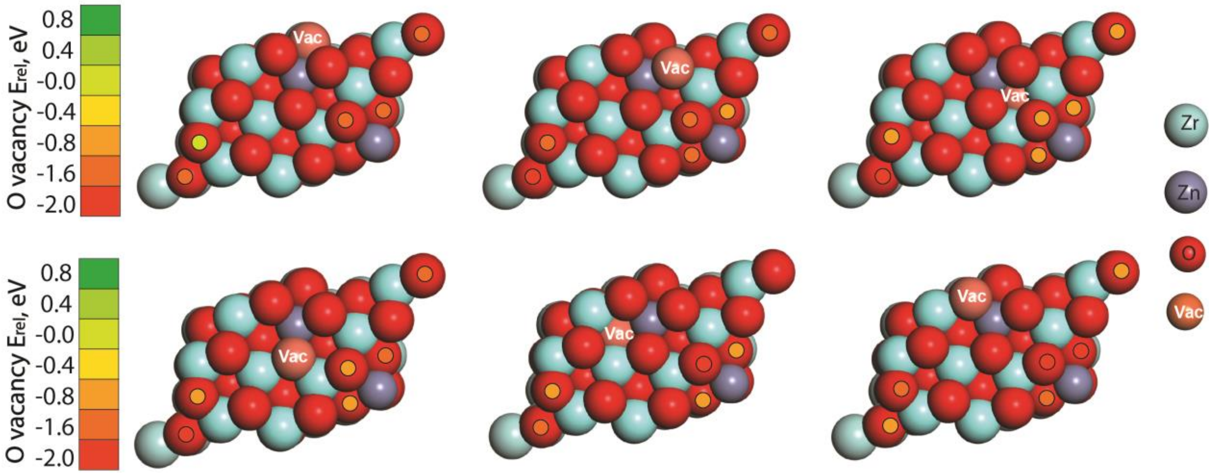

Supplementary Figure 22. Relative DFT energies (E_{rel}) of $Zn_2Zr_{n-2}O_{2n-2}$ structure with two O vacancies per unit cell of a model with Zn–Zn distance of 6.275 Å, formed via substitution of two Zn atoms to Zr atoms to acquire 1:4 Zn to Zr concentration on the surface. The second O vacancy is generated near Zn atom with respect to first O vacancy highlighted as “Vac” positions in these structures. Note that O vacancies are created near Zn atoms.

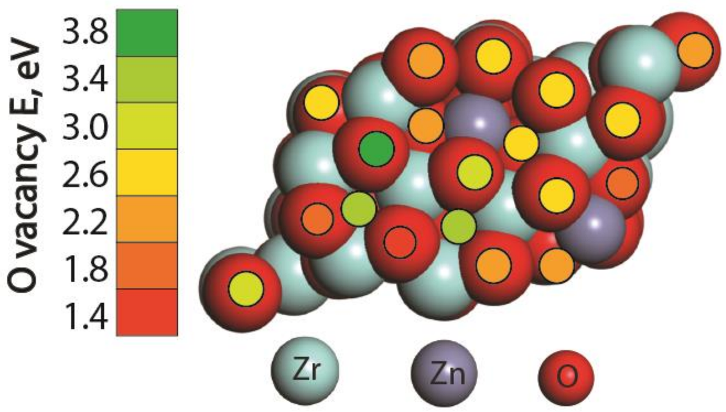

Supplementary Figure 23. Oxygen vacancy formation energy for the third vacancy per unit cell. Different O positions including top and subsurface atoms were removed to generate $Zn_2Zr_{n-2}O_{2n-3}$ model.

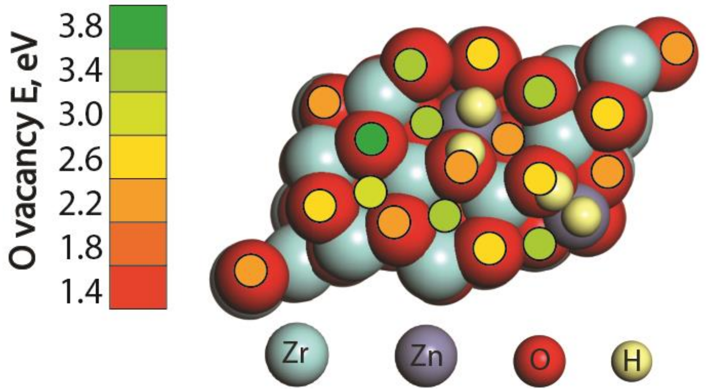

Supplementary Figure 24. Oxygen vacancy formation energy for the third vacancy per unit cell in the presence of H atoms adsorbed on the surface. Different O positions including top and subsurface atoms were removed to scan the $Zn_2Zr_{n-2}O_{2n-3}H_4$ surface. In these calculations O atoms bound to surface H were removed as OH groups.

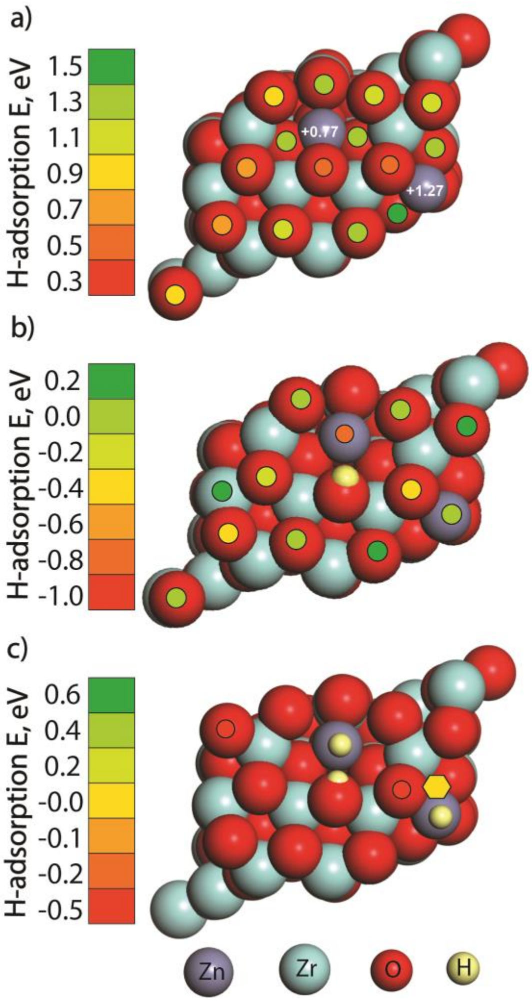

Supplementary Figure 25. DFT adsorption energy of hydrogen on $Zn_2Zr_{n-2}O_{2n-2}$ **surface. a** Adsorption of a single H atom. **b** Dissociative adsorption of H_2, and **c** adsorption of the fourth H atom produced by dissociative adsorption of the second H_2 molecule on $Zn_2Zr_{n-2}O_{2n-2}$ (101) surface. Homolytic dissociative adsorption of H_2 molecule on 2 O atoms near Zn atom with DFT energy of 0.08 eV is illustrated by the hexagon in figure **c**.

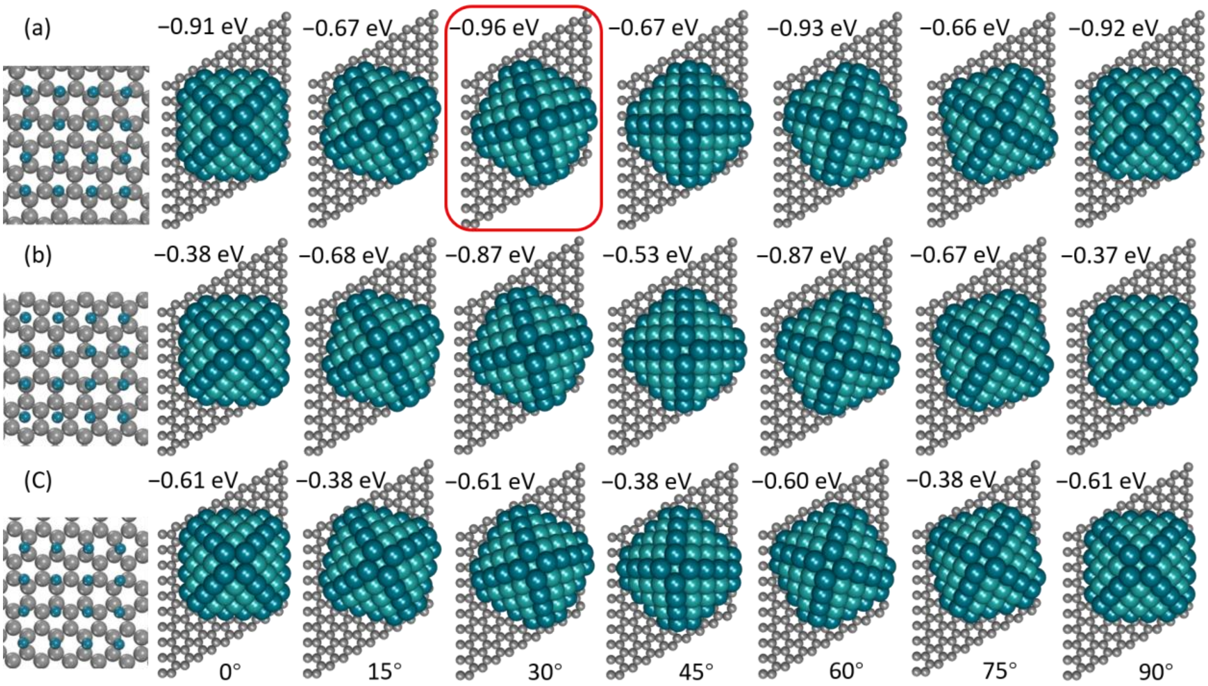

Supplementary Figure 26. Structure of Pd_{127} nanoparticles on graphene. Binding energies of Pd_{127} particles on graphene at different adsorption sites with different rotation angles around the normal to graphene passing through the center of mass of the particle, E_b = E[Pd/graphene] – E[graphene] – E[Pd]. Images on the left illustrate centering of the center of mass of Pd nanoparticle at **a** top, **b** bridge, and **c** hollow C sites corresponding to 0 degrees (Grey: C and turquoise: Pd). The most stable structure considered in further studies is highlighted by the red rectangle.

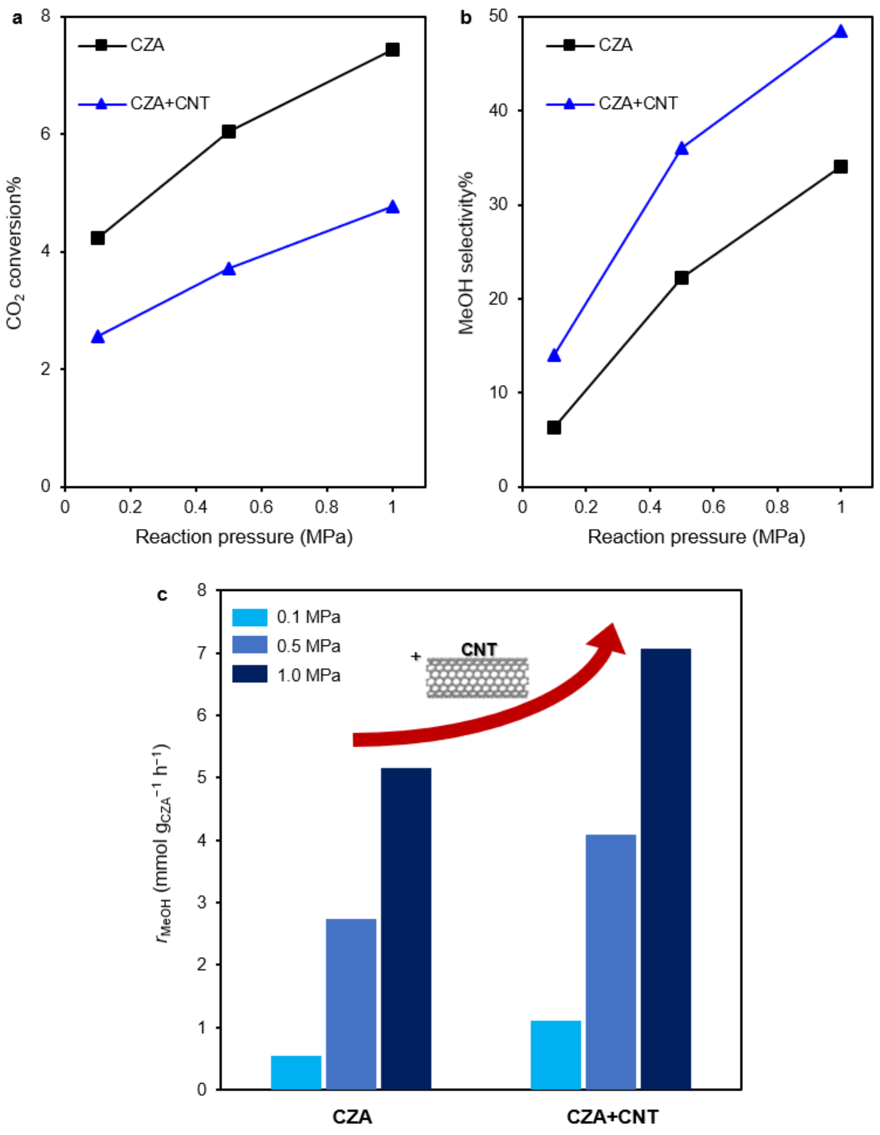

Supplementary Figure 27. Reaction data for commercial Cu/ZnO/Al_2O_3 catalyst (CZA) versus Cu/ZnO/Al_2O_3 physically mixed with CNT (CZA+CNT, CZA:CNT = 2:1 mass ratio). a CO_2 conversion, **b** MeOH selectivity, and **c** r_{MeOH} (mmol g_{CZA}^{-1} h^{-1}) as a function of reaction pressure.

3. Identify kinetics-controlled region

We tested CO_2 hydrogenation over $ZnZrO_x$ catalyst by varying GHSV under fixed temperature (533 K), pressure (5 MPa), and feed composition ($CO_2/H_2/Ar$ = 19/76/5). The CO_2 consumption rate (mmol $g_{cat.}^{-1}$ h^{-1}) is consistent under the condition where GHSV ≥ 18000 cm^3_{STP} $g_{cat.}^{-1}$ h^{-1} (Supplementary Fig. 28b). When plotting the reaction rate as a function of CO_2 conversion, the corresponding region is CO_2 conversion ≤ 1.5% (Supplementary Fig. 28c), although when CO_2 conversion falls below 0.5% expeimental error is large (i.e. carbon balance < 90%). Consequently, the activity of catalysts was determined in the conversion range of 0.5–1.4% region (see Supplementary Table 2). This conversion level is far below the thermodynamic equilibrium CO_2-to-MeOH conversion at the given condition (533 K, 5 MPa, H_2/CO_2 = 4, *ca*. 24%).

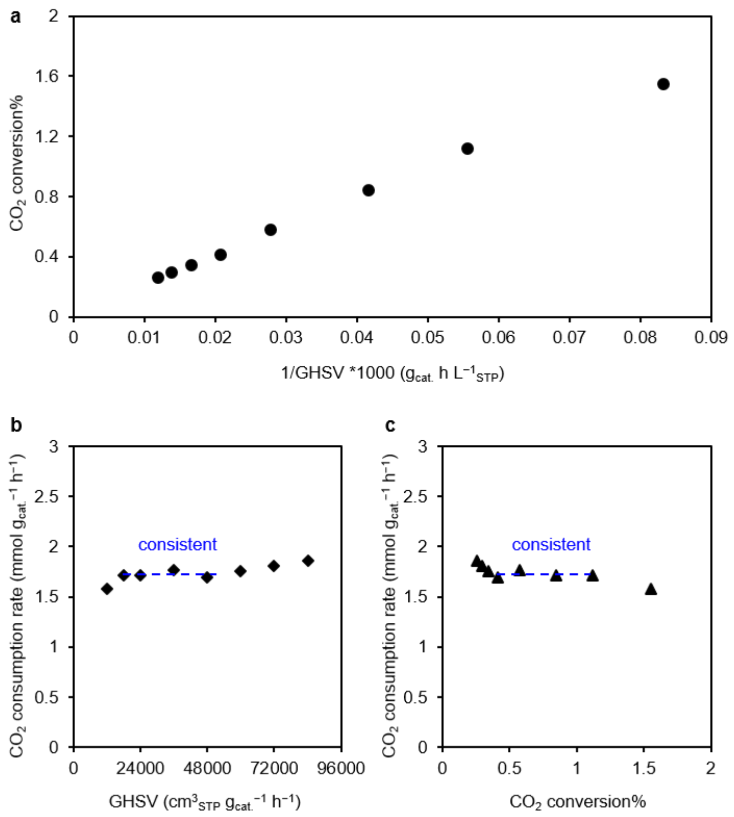

Supplementary Figure 28. Identify kinetics-controlled region for $ZnZrO_x$. a CO_2 conversion as a function of 1/GHSV. **b** CO_2 consumption rate as a function of GHSV. **c** CO_2 consumption rate as a function of CO_2 conversion (Condition: 533 K, 5 MPa, $CO_2/H_2/Ar$ = 19/76/5).

The effect of external diffusion was tested by simultaneously varying the flow rate and catalyst mass in a fixed GHSV for three catalysts, i.e. $ZnZrO_x$, Pd/$ZnZrO_x$, and Pd/CNT+$ZnZrO_x$. $ZnZrO_x$ and Pd/$ZnZrO_x$ show consistent r_{MeOH} regardless of flow rate and catalyst mass, demonstrating under the testing condition (flow rate ≥ 20 cm^3_{STP} min^{-1} and catalyst mass ≥ 0.05 g) the system is not limited by the external mass transfer (Supplementary Fig. 29a,b). Pd/CNT+$ZnZrO_x$ shows a gradual increase of r_{MeOH} under low flow condition (flow rate ≤ 96 cm^3_{STP} min^{-1} and catalyst mass ≤ 0.04 g), but exhibits consistent r_{MeOH} at higher flow rate and more amount of catalyst mass

condition (Supplementary Fig. 29c). Therefore, we used 0.05 g of catalyst and 120 cm^3_{STP} min^{-1} of flow rate to test Pd/CNT+$ZnZrO_x$.

We also compared the effect of internal diffusion for three catalysts, $ZnZrO_x$, Pd/$ZnZrO_x$, and Pd/CNT+$ZnZrO_x$, by varying pellet sizes (180–450 μm v.s. 450–850 μm). All catalysts show consistent r_{MeOH}, thus the internal diffusion limitation can be ignored in the given pellet sizes (Supplementary Fig. 30).

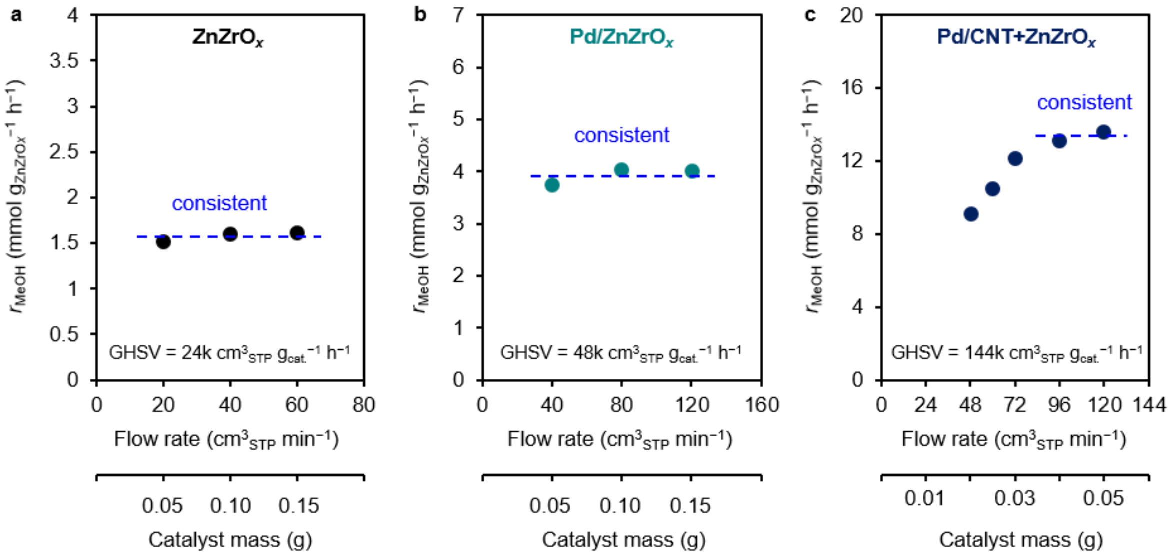

Supplementary Figure 29. Evaluation of external mass transfer limitation. The effect of flow rate and catalyst mass in a fixed GHSV on the reaction rate (r_{MeOH}) of **a** $ZnZrO_x$, **b** Pd/$ZnZrO_x$, and **c** Pd/CNT+$ZnZrO_x$ (533 K, 5 MPa, CO_2/H_2/Ar=19/76/5).

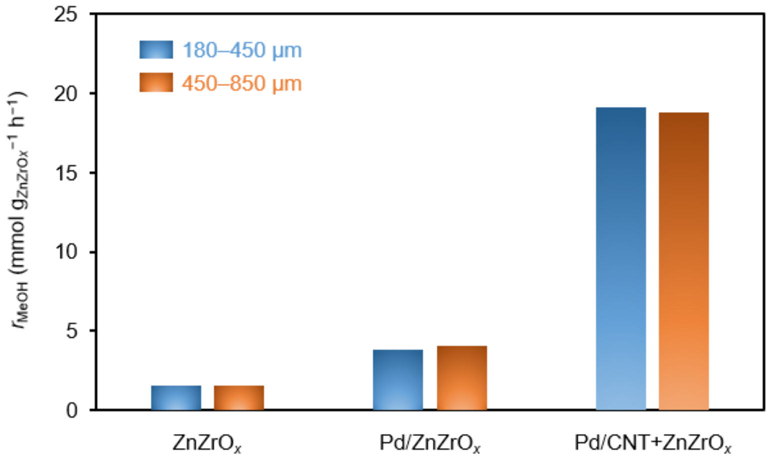

Supplementary Figure 30. Evaluation of the internal mass transfer limitation. The effect of pellet sizes on the activity of $ZnZrO_x$, Pd/$ZnZrO_x$, and Pd/CNT+$ZnZrO_x$ (533 K, 5 MPa, CO_2/H_2/Ar=19/76/5, GHSVs are shown in Supplementary Table 2).

4. Estimation of interfacial area of Pd and $ZnZrO_x$ in Pd/$ZnZrO_x$

To estimate the interfacial area of Pd with $ZnZrO_x$ in Pd/$ZnZrO_x$, we assumed that semispherical Pd particles of face-centered cubic structure (atomic packing factor = 0.74) and diameters of 5 nm are uniformly dispersed on the surface of $ZnZrO_x$ (1 wt% of Pd in the catalyst, Supplementary Table 1). $N_{\mathrm{Pd,\ 5nm}}$ which represents the number of Pd atoms in a 5 nm cluster can be obtained as follows:

$$N_{\mathrm{Pd,\ 5\ nm}} = 0.5 \times \frac{V_{5\ nm}}{V_{atom}} = 0.5 \times \frac{0.74 \times \frac{1}{6}\pi(d_{5\ nm})^3}{\frac{1}{6}\pi(d_{\mathrm{atom}})^3} = 0.5 \times \frac{0.74 \times \frac{1}{6}\pi(5\ \mathrm{nm})^3}{\frac{1}{6}\pi(0.274\ \mathrm{nm})^3} = 2248\ (\mathrm{atoms}) \quad (15)$$

In turn, $A_{\mathrm{Pd,\ 5\ nm}}$ which represents the contact area of a single 5 nm Pd particle with support can be obtained as follows:

$$A_{\mathrm{Pd,\ 5\ nm}} = \pi r^2 = \pi(\frac{\mathrm{d}}{2})^2 = \pi(2.5\ \mathrm{nm})^2 = 19.6\ \mathrm{nm}^2 \quad (16)$$

Finally, the interfacial area between Pd particles and $ZnZrO_x$ can be obtained by the following equation:

$$\text{Interfacial area} = \frac{N_{Pd,\ total}}{N_{Pd,\ 5\ nm}} \times A_{\mathrm{Pd,\ 5\ nm}} = \frac{0.01\ g_{Pd}\ g_{cat.}^{-1} \times 106.42^{-1}\ mol\ g_{Pd}^{-1} \times N_A\ atoms\ mol^{-1}}{2248\ atoms} \times 19.6\ \mathrm{nm}^2, \quad (17)$$

where N_A means Avogadro number.

The interfacial area is estimated to be 0.49 $m^2\ g^{-1}$. This is about 1% of the surface area of the catalyst (50 $m^2\ g^{-1}$, Supplementary Table 1).

5. Effects of mortar grinding on ZnZrO$_x$

In order to check the effect of mortar grinding on ZnZrO$_x$, we compared BET surface area and catalytic activity of unground ZnZrO$_x$ and mortar ground ZnZrO$_x$ (i.e. a standard catalyst in the study). As ZnZrO$_x$ is intrinsically a lump, the catalyst was just sieved without any physical grinding and pelletizing during the preparation step of catalytic reaction. As shown in Supplementary Fig. 31, mortar grinding slightly reduces surface area from 44 to 42 m^2 g^{-1}, thus it is not beneficial for improving gas diffusion to the active site of ZnZrO$_x$. Two catalysts also show identical activity within the error range.

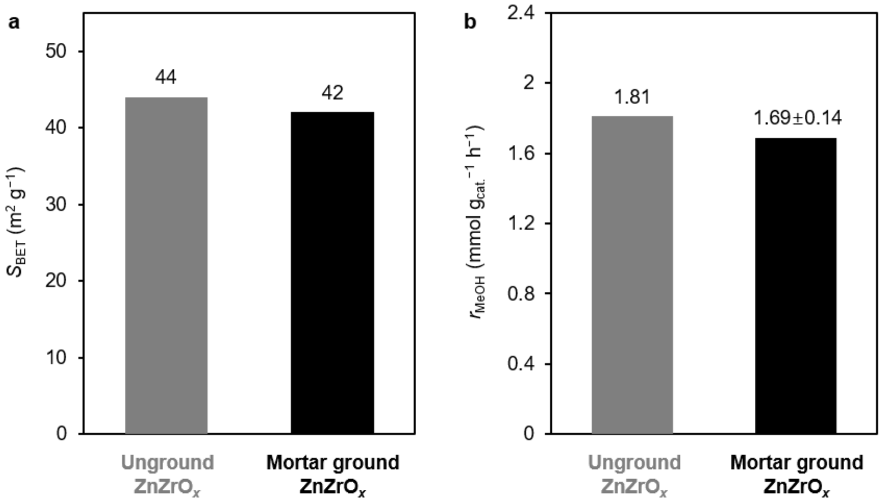

Supplementary Figure 31. Effects of mortar grinding on ZnZrO$_x$. (a) BET surface area and (b) methanol formation activity of unground and mortar ground ZnZrO$_x$ catalysts (Condition: 533 K, 5 MPa, and CO_2/H_2/Ar = 19/76/5, GHSV = 24000 cm$^3_{STP}$ g$_{cat.}^{-1}$ h^{-1}).

6. References

1 Prelazzi, G., Cerboni, M. & Leofanti, G. Comparison of H_2 adsorption, O_2 adsorption, H_2 titration, and O_2 titration on supported palladium catalysts. *J. Catal.* **181**, 73–79 (1999)

2 Canton, P. *et al.* Pd/CO average chemisorption stoichiometry in highly dispersed supported Pd/γ-Al_2O_3 catalysts. *Langmuir* **18**, 6530–6535 (2002)

3 Kwon, H. C. *et al.* Catalytic Interplay of Ga, Pt, and Ce on the Alumina Surface Enabling High Activity, Selectivity, and Stability in Propane Dehydrogenation. *ACS Catal.* **11**, 10767–10777 (2021)

4 Makuła, P., Pacia, M. & Macyk, W. How to correctly determine the band gap energy of modified semiconductor photocatalysts based on UV–Vis spectra. *J. Phys. Chem. Lett.* **9**, 6814–6817 (2018)

5 Li, X. *et al.* Determination of band gaps of self-assembled carbon nanotube films using Tauc/Davis–Mott model. *Appl. Phys. A* **97**, 341–344 (2009)

6 Wang, J. *et al.* A highly selective and stable ZnO-ZrO_2 solid solution catalyst for CO_2 hydrogenation to methanol. *Sci. Adv.* **3**, e1701290 (2017)

7 Igawa, N. & Ishii, Y. Crystal structure of metastable tetragonal zirconia up to 1473 K. *J. Am. Ceram. Soc.* **84**, 1169–1171 (2001)

8 Olah, G. A., Goeppert, A. & Prakash, G. K. S. Chemical Recycling of Carbon Dioxide to Methanol and Dimethyl Ether: From Greenhouse Gas to Renewable, Environmentally Carbon Neutral Fuels and Synthetic Hydrocarbons. *J. Org. Chem.* **74**, 487–498 (2009)

9 Shao, L. *et al.* The role of palladium dynamics in the surface catalysis of coupling reactions. *Angew. Chem. Int. Ed.* **52**, 2114–2117 (2013)

10 Huang, X. *et al.* Carbon Nanotube-Encapsulated Noble Metal Nanoparticle Hybrid as a Cathode Material for Li-Oxygen Batteries. *Adv. Funct. Mater.* **24**, 6516–6523 (2014)

11 Yang, F. H., Lachawiec, A. J. & Yang, R. T. Adsorption of spillover hydrogen atoms on single-wall carbon nanotubes. *J. Phys. Chem. B* **110**, 6236–6244 (2006)

12 Han, Z. *et al.* CO_2 hydrogenation to methanol on ZnO-ZrO_2 solid solution catalysts with ordered mesoporous structure. *J. Catal.* **396**, 242–250 (2021)

13 Zhou, C. *et al.* Highly active ZnO-ZrO_2 aerogels integrated with H-ZSM-5 for aromatics synthesis from carbon dioxide. *ACS Catal.* **10**, 302–310 (2019)

14 Wang, X. *et al.* A novel microreaction strategy to fabricate superior hybrid zirconium and zinc oxides for methanol synthesis from CO_2. *Appl. Catal. A: Gen.* **595**, 117507 (2020)

15 Lee, K. *et al.* Atomic Pd-promoted $ZnZrO_x$ solid solution catalyst for CO_2 hydrogenation to methanol. *Appl. Catal. B: Environ.* **304**, 120994 (2022)

16 Huang, C. *et al.* CO2 Hydrogenation to Methanol over PdZnZr Solid Solution: Effects of the PdZn Alloy and Oxygen Vacancy. *ACS Appl. Energy Mater.* **4**, 9258–9266 (2021)

17 Xu, D., Hong, X. & Liu, G. Highly dispersed metal doping to ZnZr oxide catalyst for CO_2 hydrogenation to methanol: Insight into hydrogen spillover. *J. Catal.* **393**, 207–214 (2021)

18 Ruland, H. *et al.* CO_2 hydrogenation with $Cu/ZnO/Al_2O_3$: A Benchmark Study. *ChemCatChem* **12**, 3216–3222 (2020)

19 Martin, O. *et al.* Indium Oxide as a Superior Catalyst for Methanol Synthesis by CO_2 Hydrogenation. *Angew. Chem. Int. Ed.* **55**, 6261–6265 (2016)

20 Liang, B. *et al.* Investigation on Deactivation of $Cu/ZnO/Al_2O_3$ Catalyst for CO_2 Hydrogenation to Methanol. *Ind. Eng. Chem. Res.* **58**, 9030–9037 (2019)

21 Wang, Y. *et al.* Exploring the ternary interactions in Cu–ZnO–ZrO_2 catalysts for efficient CO_2 hydrogenation to methanol. *Nat. Commun.* **10**, 1–10 (2019)

22 Li, M. M.-J., Zeng, Z., Liao, F., Hong, X. & Tsang, S. C. E. Enhanced CO_2 hydrogenation to methanol over CuZn nanoalloy in Ga modified Cu/ZnO catalysts. *J. Catal.* **343**, 157–167 (2016)

23 Wang, Y. *et al.* Strong evidence of the role of H_2O in affecting methanol selectivity from CO_2 hydrogenation over Cu-ZnO-ZrO_2. *Chem* **6**, 419–430 (2020)

24 Dang, S. *et al.* Rationally designed indium oxide catalysts for CO_2 hydrogenation to methanol with high activity and selectivity. *Sci. Adv.* **6**, eaaz2060 (2020)

25 Wang, J. *et al.* High-Performance MaZrOx (Ma = Cd, Ga) Solid-Solution Catalysts for CO_2 Hydrogenation to Methanol. *ACS Catal.* **9**, 10253–10259 (2019)

26 Sha, F. *et al.* The promoting role of Ga in ZnZrO*x* solid solution catalyst for CO_2 hydrogenation to methanol. *J. Catal.* **404**, 383–392 (2021)

27 Frei, M. S. *et al.* Atomic-scale engineering of indium oxide promotion by palladium for methanol production via CO_2 hydrogenation. *Nat. Commun.* **10**, 3377 (2019)

28 Rui, N. *et al.* CO_2 hydrogenation to methanol over Pd/In_2O_3: effects of Pd and oxygen vacancy. *Appl. Catal. B: Environ.* **218**, 488–497 (2017)

29 Shen, C. *et al.* Highly active Ir/In_2O_3 catalysts for selective hydrogenation of CO_2 to methanol: experimental and theoretical studies. *ACS Catal.* **11**, 4036–4046 (2021)

30 Rui, N. *et al.* Hydrogenation of CO_2 to Methanol on a $Au^{\delta+}$–In_2O_{3-x} Catalyst. *ACS Catal.* **10**, 11307–11317 (2020)

31 Han, Z., Tang, C., Wang, J., Li, L. & Li, C. Atomically dispersed Pt^{n+} species as highly active sites in Pt/In_2O_3 catalysts for methanol synthesis from CO_2 hydrogenation. *J. Catal.* **394**, 236–244 (2021)

32 Meng, C. *et al.* Oxygen-deficient metal oxides supported nano-intermetallic $InNi_3C_{0.5}$ toward efficient CO_2 hydrogenation to methanol. *Sci. Adv.* **7**, eabi6012 (2021)

33 Bavykina, A. *et al.* Turning a Methanation Co Catalyst into an In–Co Methanol Producer. *ACS Catal.* **9**, 6910–6918 (2019)

34 Nelson, J. XANES reflects coordination change and underlying surface disorder of zinc adsorbed to silica. *J. Synchrotron. Rad.* **28**, 1119–1126 (2021)
